# Spatial diarrheal disease risks and antibiogram diversity of diarrheagenic *Escherichia coli* in selected access points of the Buffalo River, South Africa

**DOI:** 10.1371/journal.pone.0288809

**Published:** 2023-08-24

**Authors:** Chidozie Declan Iwu, Nolonwabo Nontongana, Chinwe Juliana Iwu-Jaja, Brilliance Onyinyechi Anyanwu, Erika du Plessis, Lise Korsten, Anthony Ifeanyin Okoh

**Affiliations:** 1 SAMRC Microbial Water Quality Monitoring Centre, University of Fort Hare, Alice, South Africa; 2 Applied and Environmental Microbiology Research Group (AEMREG), University of Fort Hare, Alice, South Africa; 3 Department of Global Health, Stellenbosch University, Stellenbosch, South Africa; 4 Centre for Occupational Health, Safety and Environment, University of Port Harcourt, Port Harcourt, Rivers State, Nigeria; 5 Department of Plant and Soil Sciences, University of Pretoria, Hatfield, South Africa; 6 Department of Science and Technology-National Research Foundation Centre of Excellence in Food Security, Pretoria, South Africa; Beni Suef University Faculty of Veterinary Medicine, EGYPT

## Abstract

Freshwater sources, often used for domestic and agricultural purposes in low- and middle-income countries are repositories of clinically significant bacterial pathogens. These pathogens are usually diversified in their antibiogram profiles posing public health threats. This study evaluated the spatial diarrhoeal disease risk and antibiogram diversity of diarrheagenic *Escherichia coli* (DEC) in four access points of the Buffalo River, Eastern Cape Province, South Africa using standard epidemiological, culture, and molecular methods. The diarrhoeal disease risk was characterised using the Monte Carlo simulation, while the antibiogram diversity was assessed using the species observed Whittaker’s single alpha-diversity modelling. *E*. *coli* mean count was highest in King William’s Town dam [16.0 × 10^2^ CFU/100ml (SD: 100.0, 95% CI: 13.5 × 10^2^ to 18.5 × 10^2^)]. Enterohemorrhagic *E*. *coli* (*stx*1/*stx*2) was the most prevalent DEC pathotype across the study sites. A high diarrhoeal disease risk of 25.0 ×10^−2^ exceeding the World Health Organization’s standard was recorded across the study sites. The average single and multiple antimicrobial resistance indices of the DEC to test antimicrobials were highest in the Eluxolzweni dam [0.52 (SD: 0.25, 95% CI: 0.37 to 0.67)] and King William’s Town dam [0.42 (SD: 0.25, 95% CI: 0.27 to 0.57)] respectively. The prevalent antibiotic resistance genes detected were *tet*A, *bla*_FOX_ and *bla*_MOX_ plasmid-mediated *Amp*C, *bla*_TEM_ and *bla*_SHV_ extended-spectrum β-lactamases, which co-occurred across the study sites on network analysis. The phenotypic and genotypic resistance characteristics of the DEC in Maden dam (r = 0.93, *p*<0.00), Rooikrantz dam (r = 0.91, *p*<0.00), King William’s Town dam (r = 0.83, *p =* 0.0), and Eluxolzweni dam (r = 0.91, *p*<0.00) were strongly correlated. At least, three phylogenetic clades of the DEC with initial steep descent alpha-diversity curves for most of the test antimicrobials were observed across the study sites, indicating high diversity. The occurrence of diversified multi drug resistant DEC with diarrhoeal disease risks in the Buffalo River substantiates the role surface water bodies play in the dissemination of drug-resistant bacterial pathogens with public health implications.

## 1. Introduction

Globally, diarrhoeal disease has significantly caused mortality and morbidity, especially among children and neonates. In 2010, about 1.7 billion diarrhoeal cases were reported nationwide, of which 700 000 deaths among children less than five years old were reported. [1]. Diarrhoea was tagged the 5^th^ leading cause of death among children under five years of age with an estimate of 446 000 deaths and 8^th^ leading cause of death across all age groups with an estimate of 1 655 944 mortalities in 2016 [2]. Environmental factors such as contaminated and open water sources, poor sanitation, and inadequate waste disposal are the leading contributors to the high prevalence of diarrhoeal disease, especially in developing countries [3]. With South Africa being a semi-arid country, water resources are scanty. Water is often sourced from limited surface water bodies such as rivers, dams and streams [4]. This poses diarrhoeal disease risks as these water bodies are often polluted by poorly managed wastewater and uncontrolled sewage discharges [5].

The moderate to severe forms of diarrhoea in both community and clinical settings in developing countries are frequently caused by diarrhoeagenic *Escherichia coli* (DEC) [6]. Based on their phenotypic and virulence traits, the DEC has been categorised into six pathogenic groups. They include Enterohemorrhagic *E*. *coli* (EHEC) or Shiga-toxin producing *E*. *coli* (STEC), Enteroaggregative *E*. *coli* (EAEC), Enteroinvasive *E*. *coli* (EIEC), Enteropathogenic *E*. *coli* (EPEC), Enterotoxigenic *E*. *coli* (ETEC) and diffusely adherent *E*. *coli* (DAEC) [7]. Of these, EPEC, EAEC and ETEC are the most significant DEC and cause 30 to 40% of acute paediatric diarrhoea globally [8]. EHEC causes bloody diarrhoea and potential kidney complications by producing Shiga toxin, and EAEC forms biofilms and adheres to the intestinal mucosa, causing persistent watery diarrhoea. EIEC invades intestinal cells, leading to inflammation and ulceration, resulting in bloody diarrhoea and EPEC induces diarrhoea through the formation of "attaching and effacing" lesions on intestinal cells. ETEC produces enterotoxins that disrupt fluid balance in the intestine, leading to watery diarrhoea. Although, the pathogenicity of DAEC are not fully elucidated, they exhibits a diffuse adherence pattern to intestinal cells, contributing to diarrhoea [9, 10].

The infections caused by the pathogenic *E*. *coli* strains is becoming difficult to treat due to antimicrobial resistance (AMR) [10]. The presence of AMR in enteric pathogens including DEC against first-line antibiotics such as penicillins and trimethoprim-sulfamethoxazole in Africa is well recognised [11]. Resistance against multiple classes of antimicrobials is also becoming very common as transmissible ‘mobile genetic elements’ such as integrons and plasmids contain multiple gene cassettes and resistance genes encoding resistance against multiple antibiotics [12]. Since AMR is a complex public health issue, curbing it requires a multifaceted approach which uses the ‘One Health’ strategy. Unfortunately, more attention has been placed on the animal and human aspects compared to the environmental aspect [13]. To beef up the environmental AMR surveillance, more studies on AMR occurrence in the environment particularly the aquatic ecosystem is needed.

The main goal of this study was to assess the spatial diarrhoeal disease risks and antibiogram diversities of DEC recovered from selected access points of the Buffalo River, Eastern Cape Province of South Africa. Although a study was conducted in 2013 to assess the bacteriological (*Enterococci*, faecal and total coliform counts) and virological quality of the Buffalo River [14], our report, to the best of our knowledge, is the first that elucidated the prevalence, virulence, quantitative diarrhoeal disease risks and antibiogram diversities of DEC retrieved from selected access points of the river.

## 2. Methods

### 2.1 Study design and description of the study area

**Fig 1:**
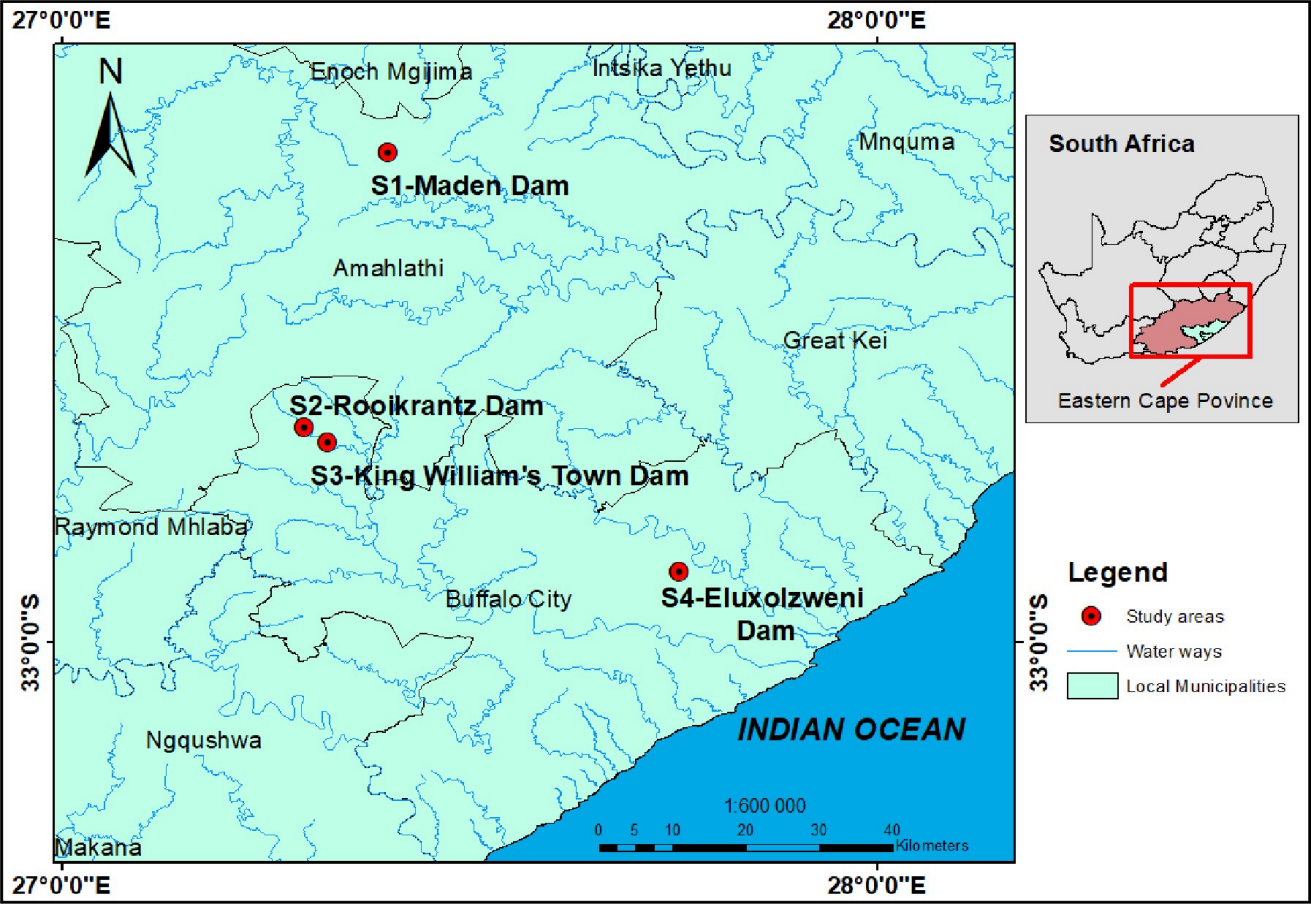
Map showing the selected access points of the Buffalo River studied.

Using epidemiological, microbiological, and molecular methods, we conducted a laboratory-based cross-sectional study to spatially evaluate the prevalence, virulence, diarrheal disease risks, antibiogram characteristics and diversities of DEC in four selected access points of the Buffalo River, Eastern Cape Province, South Africa as shown in Fig 1. The Buffalo River is the only navigable river in South Africa which serves as a principal source of water for urban, rural, and industrial consumers. It is usually used for domestic, irrigation and recreational purposes. It is sourced from the Eastern Cape Amothola Mountains and flows for 126 km South-East into the Indian Ocean at East London harbour [14]. During the flow, the Buffalo River meets the Maden Dam after 7 km from its source. Four kilometers downstream of Maden Dam is the much larger Rooikrantz Dam which flows into King William’s Town Dam. Industrial effluents, solid wastes, raw sewage, runoffs and wastewater treatment effluents from agricultural and urban areas are discharged into the river via three major tributaries including Ngqokweni, Yellow Woods River and Mgqakwebe [15]. Buffalo river provides raw water for portable water production [16], domestic activities, extensive agricultural activities, subsistence farming, irrigation, and fishing and serves as a site for tourist attractions [14] (Fig 1).

### 2.2 Field sampling

Four access points of the Buffalo River (Fig 1) including the Maden, Rooikrantz, King William’s Town and Eluxolzweni dams were selected as the study sites and field sampling points. These dams were selected due to their accessibility and proximity to human settlements and farming communities. The anthropogenic activities observed at these sites during the sampling regime are detailed in S1 Table in S1 File. Adapting a grab sampling technique, water samples (approximately 1 Litre) were collected from the selected access points of the Buffalo River using sterile polypropylene sampling bottles. This was done in triplicates to validate our empirical data and observed results between June and July 2020 during the Winter season. All the collected samples were stored on ice in a cooler box. Within 4 hours, they were transported to the laboratory for microbiological and molecular analysis.

### 2.3 Quantification and isolation of presumptive *E*. *coli*

The quantification of the *E*. *coli* was done using a three-fold (1.0 E + 01 to 1.0 E + 03) serial dilution and membrane filtration technique as previously described [17]. Assisted by a vacuum pump, 100 ml of each of the dilutions of the water samples were filtered through 0.45mm pore membrane filters. The membrane filters were aseptically placed on *E*. *coli* chromogenic agar (ECA) (Merck) plates for the enumeration and isolation of presumptive *E*. *coli* isolates. All the ECA plates were incubated in inverted positions at 37°C for 24 hours as recommended by the manufacturers of the media. After incubation, blue colonies which are presumptive of *E*. *coli* on the ECA were enumerated and the data were transformed to CFU/100ml of the water samples analysed. To obtain pure culture of *E*. *coli* isolates, the presumptive isolates were sub-cultured on Eosin Methylene Blue agar (EMBA) (Merck, South Africa) and incubated at 37°C for 24 hours. Distinct and morphologically clear isolates with green metallic sheen, were picked at random to avoid clonal similarities. They were stored at −80°C in 25% glycerol prior to molecular analysis.

### 2.4 Molecular confirmation and delineation of *E*. *coli* into diarrheagenic pathotypes

This was started off by resuscitating the isolates using Nutrient Broth (Merck) and extracting their genomic DNA using the boiling extraction method [18]. A Polymerase Chain Reaction (PCR) assay was carried out to confirm the identities of the isolates. This was done by targeting the *E*. *coli* housekeeping 4-methyl umbelliferyl-glucuronide (*uid*A) gene marker [19]. Using ‘simplex PCR’ assays and targeting specific virulence gene markers (*eagg*, *ipaH*, *daa*E, *lt*, *eae*, *bfp*, *stx1*, *stx2* detailed in S2 Table in S1 File), the confirmed *E*. *coli* isolates were delineated into six DEC strains namely: EIEC, EPEC, DAEC, EHEC EAEC and ETEC as described by Titilawo et al. (2015a) [20]. All the PCR products were electrophoresed and viewed using the ultraviolet trans-illuminator (Alliance 4.7, United States). S2 Table in S1 File details the primer sequences, expected amplicon sizes and cycling conditions used in the molecular amplification of the *uid*A and virulence genes. *E*. *coli* ATCC 25922 was included as a positive control [21].

### 2.5 Spatial diarrheal disease risk modelling of DEC

The risks of diarrhoeal disease attributed to DEC in the selected access points of the Buffalo River were evaluated using the site-based Quantitative Microbial Risk Modelling as described by the Codex Alimentarius Commission [22]. First, we characterized the hazard using the “Beta-Poisson model” (Eq 1) [23].


$$Pinf=1-1+\frac{-D}{\beta }\alpha$$ (Eq 1)

P_inf_ is the probability of infection in a person exposed to a given dose (D) of *E*. *coli* which is evaluated using Eq 2., α = 0.0571 and β = 2.2183 are the shape parameters for *E*. *coli* [24].


D=(Iv×Mc) (Eq 2)

D is *E*. *coli* ingestion dose, I_v_ is the volume of the water ingested and M_c_ is the *E*. *coli*. mean concentration.

Secondly, we carried out an exposure assessment to estimate the number of potential exposures that exist between the hazard and the population using the exposure assessment model (Eq 3) [25].


E=CR−1IM (Eq 3)

E is the Exposure parameter., C is *E*. *coli* mean concentration /100 ml of the water samples., I is the proportion of diarrhoeagenic *E*. *coli* isolates., M is the amount of water ingested per day and R is the isolation method recovery efficacy which was estimated using Eq 4 [25].


R=(Po–P/Po)×100 (Eq 4)

Po is the number of presumptive *E*. *coli* and P is the number of confirmed isolates. All the parameters inputted for exposure assessment are detailed in S3 Table in S1 File.

Lastly, we pooled the data curated from hazard characterization and exposure assessments to predict the spatial annuitized infection risk and diarrheal disease risk in the potentially exposed population using the annuitized probability of infection model (Eq 5) and the annuitized risk of illness model (Eq 6) respectively [24].


Pinf/y=1–(1–Pinf)E (Eq 5)

P_inf/y_ is the annuitized infection probability., P_inf_ is the infection probability and E is the exposure parameter.


Pill=Pinf/y×Pill/inf (Eq 6)

P_ill_ is the annuitized diarrheal disease risk., P_inf/y_ is the annuitized infection probability and P_ill/inf_ is the illness constant for pathogenic *E*. *coli* given as 0.25 [26]. The risks were characterized using the Monte Carlo simulation with 10, 000 iterations.

### 2.6 Site-based phenotypic AMR assessments of DEC

#### 2.6.1 Antimicrobial susceptibility testing of DEC

Following the description of Iwu et al. (2022) [27], the antimicrobial susceptibility profiles of each DEC retrieved from the study sites were evaluated using the disk diffusion techniques recommended by the ‘Clinical Laboratory Standards Institute (CSLI, 2018) [28] and the ‘European Committee on Antimicrobial Susceptibility Testing (EUCAST, 2018) [29]. Sixteen antibiotics normally used to treat *E*. *coli* infections were used to evaluate the antimicrobial susceptibility profiles of the DEC. These antibiotics, their classes and concentrations are listed in S4 Table in S1 File. The isolates were categorized as either Resistant (R), Intermediate (I) or Susceptible (S) depending on their response to the test antibiotics as recommended by the CSLI and EUCAST. *E*. *coli* ATCC 25922 and *S*. *aureus* ATCC 25923 were included as reference strains for quality control for the antimicrobial susceptibility tests.

#### 2.6.2 Single and multiple antimicrobial resistance indexing and phenotyping of DEC

Eq 7 was used to estimate the Single Antimicrobial Resistance Indices (SARI) of the DEC in the selected access points of the Buffalo River.


SARI=(R+I)/(R+I+S) (Eq 7)

R, I and S are the number of resistant, intermediate, and susceptible isolates respectively.

A DEC is considered multidrug-resistant (MDR) if it confers resistance against 3 or more test antimicrobial classes. Eq 8 was used to estimate the frequencies of the MDR DEC in each study site while Eq 9 was used to estimate the multiple antimicrobial resistance indices (MARI) of the DEC in each study site. The multiple antimicrobial resistance phenotypes (MARPs) of the MDR DEC were evaluated and mapped as previously described [27].


$$MDR\;(\%)=\frac{Number\;of\;MDR\;isolates}{Total\;number\;of\;isolates}\times 100$$ (Eq 8)

$$MARI=\frac{Number\;of\;antibiotics\;a\;bacteria\;is\;resistant\;against}{Total\;number\;of\;test\;antibiotics}$$ (Eq 9)

### 2.7 Site-based genotypic AMR assessments of DEC

#### 2.7.1 Molecular screening and network analysis of antimicrobial resistance genes in DEC

Putative antibiotic resistance genes (ARGs) in all the DEC recovered from the selected access points of the Buffalo River were screened using PCR assays. Nineteen non β-lactam ARGs/ESBLs encoding tetracycline, phenicol, sulfonamide and aminoglycoside resistance were screened as described by [30] while twenty β-lactam/ESBLs ARGs encoding plasmid-mediated *Amp*C β-lactamase, ESBLs and carbapenemases were screened as described by [31]. All the ARGs, the sequences of their genome, expected lengths of the amplicons and the PCR thermal conditions used in the amplification of the non β-lactam ARGs and the β-lactam ARGs are detailed in S5 and S6 Tables in S1 File respectively. The co-occurrence patterns of the ARGs and study sites were analyzed using network analysis to identify clusters of ARGs that tend to co-occur and the study sites where these clusters are prevalent. This information can contribute to understanding the spread and dissemination of antimicrobial resistance in different environments.

#### 2.7.2 Multiple antimicrobial resistance genotyping of DEC

The multiple antimicrobial resistance genotypes (MARGs) of the MDR DEC harbouring ≥3 ARGs across the selected access points of the Buffalo River were evaluated and the patterns were mapped as described by [32].

### 2.8 Assessment of the association between the phenotypic and genotypic antimicrobial resistance profiles of DEC in each study site

Pearson’s correlation statistical analysis was carried out to evaluate the association between the phenotypic and genotypic AMR profiles of the DEC in each study site. This was followed by fitting simple linear regression models to account for the correlation responses and test if the genotypic resistance determinants in the DEC significantly predicted the phenotypic resistance in each study site. As a form of model diagnostics, the goodness of fit R-squared (R^2^) statistics were evaluated to measure how much variation of the phenotypic AMR characteristics of the DEC in each study site is explained by the genotypic AMR characteristics of the isolates in the regression models.

### 2.9 Site-based evaluation of antibiogram-based diversities of DEC

To fully elucidate the antibiogram-based diversities of the DEC in each study site, we first constructed a heatmap of the antibiogram fingerprints of the DEC across the study sites. This was followed by analysing the fingerprints using a multivariate classical clustering technique to assess the relatedness of the isolates. The Jaccard similarity indices of the isolates in each study site were generated using Eq 10 [33] and visualised using a neighbour-joining dendrogram and phylogenetic clades [34]. This was validated by evaluating the diversity indices of the isolates in each study site using the species-observed Whittaker’s single alpha diversity (α-diversity) modelling as described by Wagner et al. (2018) [36]. Site-based α-diversity curves which completely portray the evenness of a bacterial community [35] were generated using the computational formula in Eq 11. A perfectly even community is represented by a horizontal line (*D* does not change as α increases) and a highly uneven community is represented by a curve with an initial steep descent as α increases [36].

$$J(A,B)=\frac{\left|A\cap B\right|}{\left|A\cup B\right|}=\frac{\left|A\cap B\right|}{\left|A\right|}+|B|-|A\cap B|$$ (Eq 10)

Where *J* is the Jaccard similarity coefficient, A and B are finite sets of antibiogram observations.

$$D(\alpha )=(\sum_{k=1}^{k}{pk}^{\alpha }{)}^{\frac{1}{1}}-\alpha$$ (Eq 11)

Where *D* (diversity) is usually calculated for *α* = 0,1,2… and *p* is a frequency function for each antimicrobial, *k*. *D* is undefined for *α* = 1, so the limit as α approaches 1 was used instead [36].

### 2.10 Statistical and spatial analysis

Univariate and inferential statistical analysis of all the primary data were done using STATA 15 (Stata Corp LLC 4905, Lakeway Drive College Station, Texas 77845, USA). The Shapiro-Wilk analysis was used to evaluate the normal distribution of the data. The null hypothesis of normality was rejected when *p*<0.05. Frequencies and appropriate measures of central tendency and dispersion with their 95% confidence intervals (CI) were used to summarize the data. The one-way analysis of variance (ANOVA) was used to compare the microbial counts, SARI and MARI of the isolates between the study sites. The Bonferroni post hoc correction analysis was used to determine the pairs that were significantly different. *P* = 0.05 (two-tailed) was set as the benchmark. The coordinate data for each sampling site was obtained on the spot using the Etek GPS instrument (EB-12A, Taiwan). All the Geographic Information Systems (GIS) shapefiles used in the study were analysed and displayed using ArcGIS (version 10.8).

## 3 Results and discussion

### 3.1 Spatial variation of presumptive *E*. *coli* counts in the selected access points of Buffalo River

**Fig 2:**
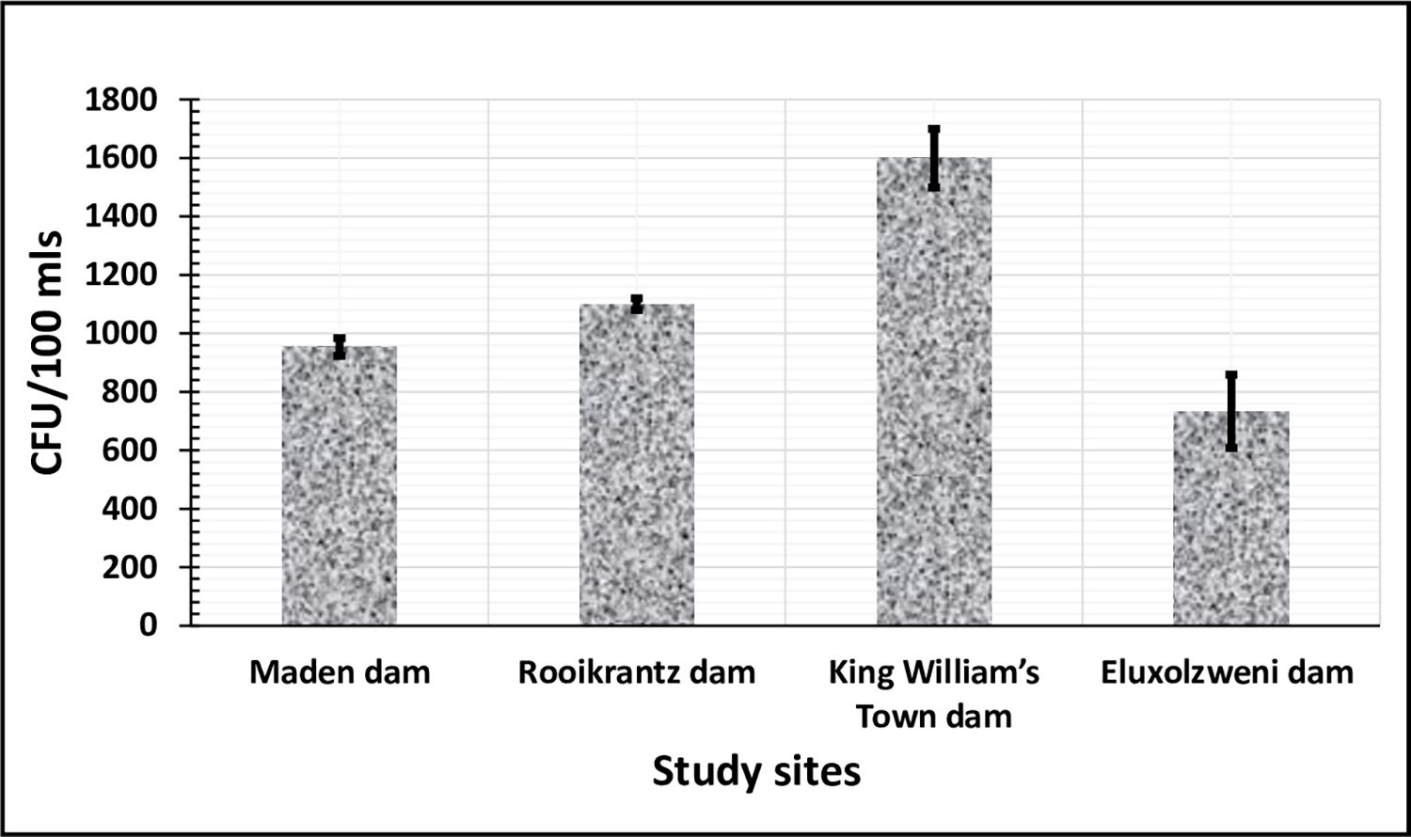
Comparative mean count of presumptive *E*. *coli* across the four study sites. The Mean ± SD *E*. *coli* counts between the different study sites were statistically different (*P* < 0.00, F = 51.62).

Fig 2 shows the comparative mean counts of presumptive *E*. *coli* in the selected four access points of the Buffalo River. The results indicated that the mean *E*. *coli* count in the study sites follow this order: 8.2 × 10^2^ CFU/100ml (SD: 125.8, 95% CI: 5.0 × 10^2^ to 11.3 × 10^2^) in Eluxolzweni dam, 9.5 × 10^2^ CFU/100ml (SD: 30.6, 95% CI: 8.8 × 10^2^ to 10.3 × 10^2^) in Maden, 11.0 × 10^2^ CFU/100ml (SD: 20.0, 95% CI: 10.5 × 10^2^ to 11.5 × 10^2^) in Rooikrantz dam and 16.0 × 10^2^ CFU/100ml (SD: 100.0, 95% CI: 13.5 × 10^2^ to 18.5 × 10^2^) in King William’s Town dam. The *E*. *coli* count in all the study sites exceeded the standard (0 CFUs /100ml or cells/100 cm^3^) set by the “Department of Water Affairs and Forestry of South Africa” for water intended for domestic, agricultural, industrial, and recreational purposes [37]. *E*. *coli* is a commonly used indicator organism for fecal pollution as it is abundant in the intestines of warm-blooded animals, including humans. High counts of *E*. *coli* suggest that the water may be contaminated with harmful pathogens, such as other bacteria, viruses, or parasites, which could pose a risk to human health upon ingestion or contact. Unless adequately treated, the use of these water sources may result in acute or chronic health issues.

Overall, the mean *E*. *coli* counts between the four study sites were found to be statistically different (*P* < 0.00, F = 51.62) following the One-way ANOVA. However, the Bonferroni post hoc correction analysis which is more specific indicated that the mean *E*. *coli* counts between Maden dam and Eluxolzweni dam (*p* = 0.46) and that between Maden dam and Rooikrantz dam (*p* = 0.37) were not significantly different. It’s not surprising that the *E*. *coli* mean count in King William’s Town dam was statistically higher than that obtained from the other study sites, as this dam was a dump site and a receiving shed of WWTP effluents.

### 3.2 Confirmation and characterization of DEC in the selected access points of the Buffalo River

**Fig 3:**
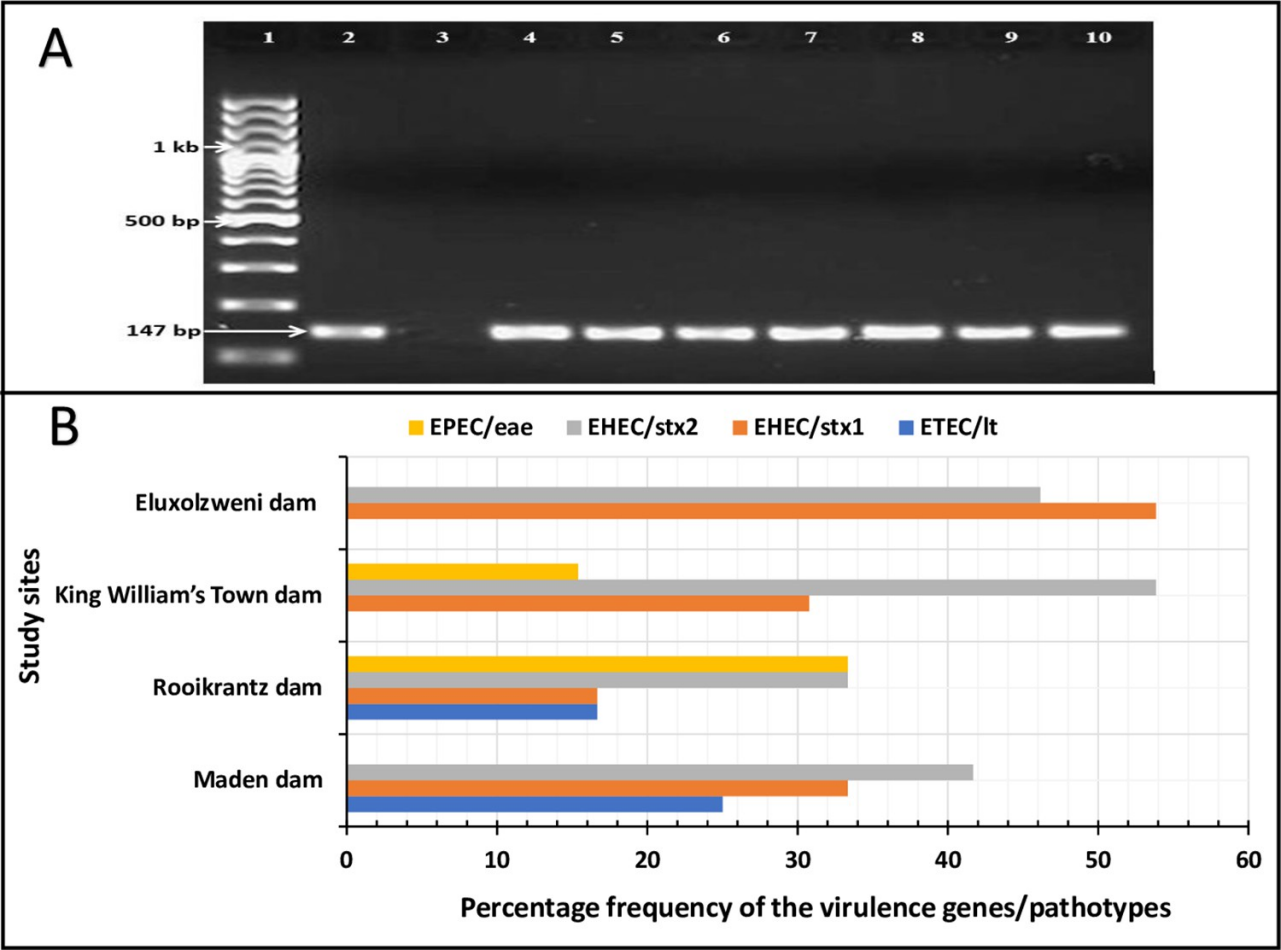
The molecular confirmation of *E*. *coli* (A) and the prevalence of DEC strains and its encoded genetic markers in the selected access points of the Buffalo River (B). Lane 1 on the gel image represents a 100 bp DNA ladder, lane 2 represents a positive control (*E*. *coli* ATCC 25922), lane 3 represent a negative control and lane 4 to 10 represent positive isolates.

Fifty presumptive *E*. *coli* isolates were recovered from each of the study sites making a total of 200 presumptive *E*. *coli* isolates. Ninety percent (180/200) of these isolates were confirmed to be *E*. *coli* as shown in the gel image in Fig 3A. Specifically, 84% (42/50), 88% (44/50), 96% (48/50) and 92% (46/50) of the confirmed *E*. *coli* isolates were retrieved from Maden dam, Rooikrantz dam, King William’s Town dam and Eluxolzweni dam; respectively. The presence of *E*. *coli* in an aquatic ecosystem is an indicator of faecal pollution which originates from humans and warm-blooded animals [38]. The high confirmation rate of *E*. *coli* across all the study sites indicates that Buffalo River is constantly exposed to sewage disposal probably from the wastewater treatment plants around its catchment making it a potential reservoir of water-borne disease pathogens.

Of the confirmed *E*. *coli* isolates, only 28% (50/180) were DEC. Specifically, 29% (12/42), 27% (12/44), 27% (13/48) and 28% (13/46) of the DEC were recovered from Maden dam, Rooikrantz dam, King William’s Town dam and Eluxolzweni dam; respectively. The DEC pathotypes and their associated virulence genes detected in the present study include ETEC/*lt*, EHEC/*stx*1, EHEC/*stx*2 and EPEC/*eae*. These pathotypes are associated with high morbidities and mortalities globally [39]. ETEC encoded by *lt* gene causes persistent and watery diarrhoea especially in immunocompromised persons, travellers, and children < 5 years. Its site of colonization is the small intestine, and the treatment is often done using rehydration and antibiotics like fluoroquinolones [40]. EHEC encoded by *stx*1 and *stx*2 genes cause watery diarrhoea, haemorrhagic colitis, and haemorrhagic uremic syndrome, especially in immunocompromised adults and children. Its site of colonization is the distal ileum and the colon, and the treatment is often done using hydration and supportive care [40]. EPEC encoded by *eae* causes profuse watery diarrhoea especially in immunocompromised adults and children < 5 years. Its site of colonization is the small intestine, and the treatment is often done using oral rehydration and antibiotics for persistent cases [40].

The prevalence of the pathotypes/virulence genes in this study ranged from 0.0% (EPEC/*eae*) to 41.7% (EHEC/*stx*2) in Maden dam, 16.7% (ETEC/*lt* and EHEC/*stx*1) to 33.3% (EHEC/*stx*2 and EPEC/*eae*) in Rooikrantz dam, 0.0% (ETEC/lt) to 53.8% (EHEC/*stx*2) in King William’s Town dam and 0.0% (EPEC/*eae* and ETEC/*lt*) to 53.8% (EHEC/*stx*1) in Eluxolzweni dam as shown in Fig 3B. The EHEC was found to be the most prevalent pathotype across all the study sites. This contrasted with a similar study in South Africa where EPEC rather than EHEC occurred the most in freshwater samples [41]. Since the primary reservoirs/sources of contamination of DEC are majorly humans, animals, food and water, the variations in the prevalence of DEC across the study sites in this study and the studies elsewhere may be attributed to the different anthropogenic activities that occur in those areas.

### 3.3 Spatial diarrheal disease risks attributed to DEC in the selected access points of the Buffalo River

The site-specific results of the hazard characterization, exposure assessments and risk characterization of the DEC in the selected access points of the Buffalo River are shown in Table 1. The daily probability of infection was lowest in Eluxolzweni dam (Mean: 37.42 × 10^−2^, Range: 36.88 × 10^−2^ to 37.97 × 10^−2^) and highest in King William’s Town dam (Mean: 39.79 × 10^−2^, Range: 39.56 × 10^−2^ to 39.99 × 10^−2^) while the exposure parameter was lowest in Maden dam (Mean: 172.79 × 10^2^, Range: 166.75 × 10^2^ to 177.63 × 10^2^) and highest in King William’s Town dam (Mean: 1080.00 × 10^2^, Range: 1012.50 × 10^2^ to 1147.50 × 10^2^). These culminated in an annual infection risk of 0.01 × 10^2^ and annual diarrhoeal disease risk of 25.0 × 10^−2^ across all the study sites. Our findings exceeded the annual infection risk standards (1.0 × 10^−4^ for drinking water and 1.0 × 10^−6^ for grey water) [42, 43] and the annual diarrhoeal disease risk standard (1.0 × 10^−3^) [43, 44] set by the World Health Organization (WHO). Similar studies recorded a lower annual infection risk that ranged from 0.90 × 10^−2^ to 0.99 × 10^−2^ attributed to *E*. *coli* O157:H7 in a lagoon and reclaimed wastewater [45] as well as a lower diarrhoeal disease risk of 4.6 × 10^−2^ attributed to *E*. *coli* O157:H7 in wastewater used for irrigation purposes [24]. This implies that the water in the selected study sites of the Buffalo River poses public health risks and caution should be taken before using the water for domestic, agricultural, or recreational purposes.

**Table 1:** Site-based estimates of diarrhoeal diseases risks attributed to DEC in the selected access points of the Buffalo River.

	Maden dam			Rooikrantz dam			King William’s Town dam			Eluxolzweni dam		
Parameters	Min	Mean	Max	Min	Mean	Max	Min	Mean	Max	Min	Mean	Max
**Hazard characterization**												
Ingestion dose (D × 10^2^)	92.00	95.33	98.00	108.00	110.00	112.00	150.00	160.00	170.00	70.00	81.66	95.00
Probability of infection/daily infection risks (P_inf_ ×10^−2^)	37.85	37.98	38.08	38.42	38.48	38.55	39.56	39.79	39.99	36.88	37.42	37.97
**Exposure assessment**												
Exposures (E × 10^2^)	166.75	172.79	177.63	243.00	247.50	252.00	1012.50	1080.00	1147.50	245.00	285.83	332.50
**Risk characterization**												
Annuitized infection risks (P_inf/y_ × 10^2^)	0.01	0.01	0.01	0.01	0.01	0.01	0.01	0.01	0.01	0.01	0.01	0.01
Annuitized diarrheal disease risks (P_ill_ ×10^−2^)	25.0	25.0	25.0	25.0	25.0	25.0	25.0	25.0	25.0	25.0	25.0	25.0

Min: Minimum, Max: Maximum

### 3.4 Phenotypic AMR characteristics of the DEC in the selected access points of the Buffalo River

#### 3.4.1 Antimicrobial susceptibility profiles of DEC

**Fig 4:**
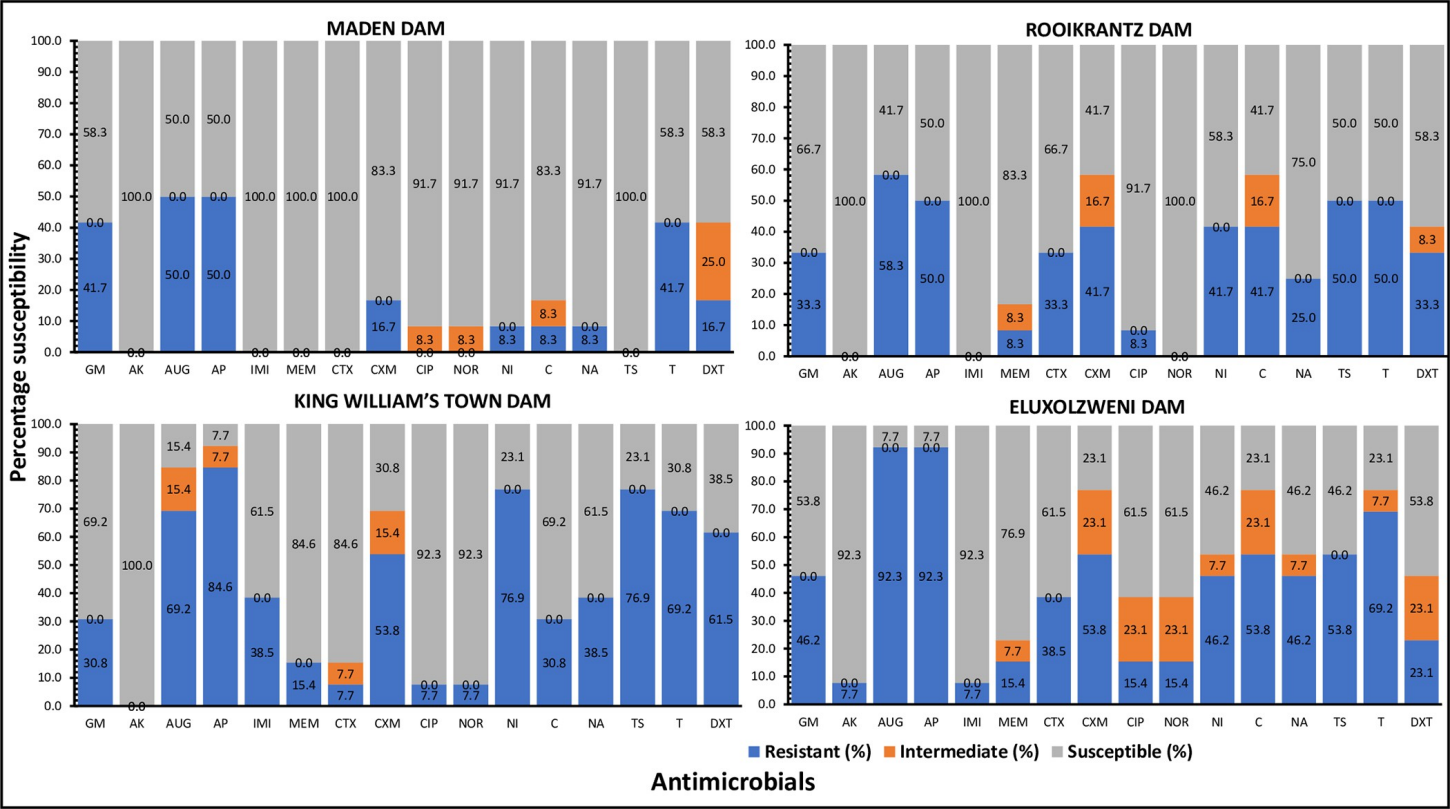
Antimicrobial susceptibility profiles of DEC across the seleceted access points of the Buffalo River. GM-gentamycin, AK-amikacin, AUG-amoxycillin-clavulanic acid, AP-ampicillin, IMI-imipenem, MEM-meropenem, CTX-cefotaxime, CXM-cefuroxime, CIP-ciprofloxacin, NOR-norfloxacin, NI-nitrofurantoin, C-chloramphenicol, NA-nalidixic acid, TS-trimethoprim/sulphamethoxazole, T-tetracycline, DXT-doxycycline.

The antimicrobial susceptibility profiles of the DEC across the four selected access points of the Buffalo River are shown in Fig 4. In Maden dam, the percentage susceptibility of DEC ranged from 50% (ampicillin and amoxycillin clavulanic acid) to 100% (cefotaxime, meropenem, amikacin, imipenem and trimethoprim/sulphamethoxazole), while the percentage resistance ranged from 0% (amikacin, imipenem, meropenem, cefuroxime, ciprofloxacin, norfloxacin and trimethoprim/sulphamethoxazole) to 50% (amoxycillin clavulanic acid and ampicillin). In Rooikrantz dam, the percentage susceptibility of DEC ranged from 41.7% (cefuroxime, chloramphenicol and amoxycillin clavulanic acid) to 100% (imipenem, amikacin and norfloxacin), while the percentage resistance ranged from 0% (amikacin, imipenem and norfloxacin) to 58.3% (amoxycillin clavulanic acid). In King William’s Town dam, the percentage susceptibility of DEC ranged from 7.7% (ampicillin) to 100% (amikacin), while the percentage resistance ranged from 0% (amikacin) to 84.6% (ampicillin). In Eluxolzweni dam, the percentage susceptibility of DEC ranged from 7.7% (ampicillin and amoxycillin clavulanic acid) to 92.3% (imipenem and amikacin), while the % resistance ranged from 7.7% (amikacin and imipenem) to 92.3% (amoxycillin clavulanic acid and ampicillin). Elevated resistance against amoxicillin clavulanic acid and ampicillin was noticed across all the study sites, corroborating previous study [46]. The high resistance levels suggest these antibiotics may be less effective in treating *E*. *coli*-related diarrheal diseases. Since the antibiotics tested in this study are used to treat infections caused by pathogenic *E*. *coli*, the susceptibility and resistance profiles observed in this study can be used as a proxy to indicate the antibiotics potentially effective for the treatment of infections caused by *E*. *coli* pathogens in the clinical settings.

#### 3.4.2 Single and multiple antimicrobial resistance indices of DEC

**Fig 5:**
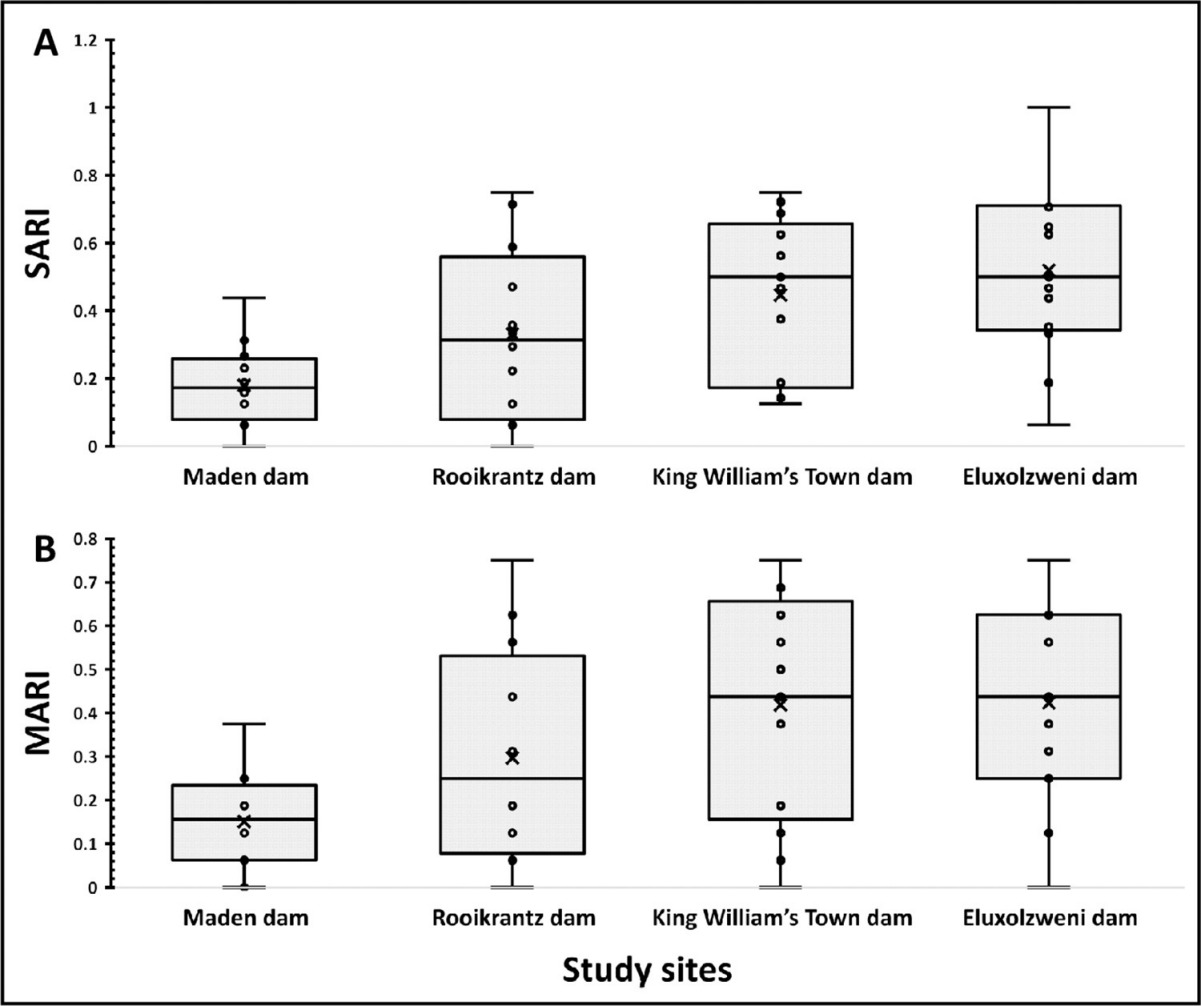
The single (A) and multiple (B) antimicrobial resistance indices of DEC across the selected access points of the Buffalo River. ^x^Mean scores, SARI: Single Antimicrobial Resistance Index, MARI: Multiple Antimicrobial Resistance Index.

The SARI and MARI are good tools for health risk assessment which suggest if bacterial isolates are from a region of high or low use of antimicrobials. The SARI of DEC across the four selected access points of the Buffalo River is shown in Fig 5A. In Maden dam, the SARI of DEC ranged from 0.06 to 0.43 with a mean score of 0.18 (SD: 0.12, 95% CI: 0.10 to 0.26), while that in Rooikrantz dam ranged from 0.00 to 0.75 with a mean score of 0.33 (SD: 0.26, 95% CI: 0.17 to 0.49). In King William’s Town dam, the SARI of DEC ranged from 0.13 to 0.75 with a mean score of 0.45 (SD: 0.23, 95% CI: 0.31 to 0.58), and that in Eloxolzweni dam ranged from 0.063 to 1.00 with a mean score of 0.52 (SD: 0.25, 95% CI: 0.37 to 0.67). The SARI values are on a spectrum of 0 to 1. Values close to 1 indicate high rate of resistance [47]. Aside from the Eluxolzweni dam, our findings indicate relatively low resistance rate in Maden, Rooikrantz and King William’s Town dams. There was an overall significant difference in the SARI of DEC across the four access points of the Buffalo River following the one-way ANOVA (*p* = 0.0025). However, on specific post hoc analysis using the Bonferroni correction test, the difference in the SARI of DEC between King William’s Town dam and Eluxolzweni dam (*p* = 1.000), Rooikrantz dam and Eluxolzweni dam (*p* = 0.240), Rooikrantz dam and King William’s Town dam (*p* = 1.000), Rooikrantz dam and Maden dam (*p* = 0.603) were not significantly different. These findings suggest that there may be variations in antimicrobial resistance levels of DEC among the access points of the Buffalo River. The lower resistance rates in Maden, Rooikrantz, and King William’s Town dams indicate a relatively lower prevalence of antimicrobial resistance in these areas, which is promising from a public health perspective. The higher resistance rates observed in Eloxolzweni dam raise concerns about potential health risks associated with antimicrobial resistance in that specific area.

The MARI of DEC across the four selected access points of the Buffalo River is shown in Fig 5B. The MARI of DEC in Maden dam ranged from 0.00 to 0.38 with a mean score of 0.15 (SD: 0.11, 95% CI: 0.08 to 0.22), while that in Rooikrantz dam ranged from 0.00 to 0.75 with a mean score of 0.30 (SD: 0.25, 95% CI: 0.14 to 0.45). The MARI of DEC in King William’s Town dam ranged from 0.00 to 0.75 with a mean score of 0.42 (SD: 0.25, 95% CI: 0.27 to 0.57), while that in Eluxolzweni dam ranged from 0.00 to 0.75 with a mean score of 0.42 (SD: 0.23, 95% CI: 0.29 to 0.56). MARI ≥ 0.2 is a threshold that indicates that the isolates are from a ‘high-risk environment’ where antimicrobials are misused [48]. Our findings, therefore, indicate that Rooikrantz, King William’s Town and Eluxolzweni dams are high-risk environments where selective pressures for AMR occur. The overall MARI of DEC were significantly different across the four access points of the Buffalo River following the one-way ANOVA (*p* = 0.0096). However, on specific post hoc analysis using the Bonferroni correction test, the difference in the MARI of DEC between King William’s Town dam and Eluxolzweni dam (*p* = 1.000), Rooikrantz dam and Eluxolzweni dam (*p* = 0.931), Rooikrantz dam and King William’s Town dam (*p* = 1.000), Rooikrantz dam and Maden dam (*p* = 0.650) were not significantly different. A possible interpretation is that there may be similar selective pressures driving antimicrobial resistance across these access points. Factors such as antibiotic usage patterns, environmental contamination, or other local factors may contribute to comparable levels of resistance.

#### 3.4.3 Multiple antimicrobial resistance phenotypes of DEC

The MARPs show the manifestation of resistance by the isolates to three or more antibiotics. About 58% (7/12) of the DEC recovered from Maden and Rooikrantz dam were multidrug-resistant against the test antimicrobials. About 77% (10/13) and 84% (11/13) of the DEC recovered from King William’s Town and Eluxolzweni dam were MDR respectively. The MARPS patterns of all the MDR DEC and their associated MARI across the study sites are shown in Table 2. The majority of the MARPS patterns occurred uniquely across all study sites. This corroborates our previous study that evaluated the MARPS patterns of pathogenic *E*. *coli* strains in surface water bodies used for irrigation purposes [27]. In the Maden dam, ‘AP-AUG-GM-T’ and ‘AP-AUG-CXM’ occurred in duplicate while in the Eluxolzweni dam, ‘AP-AUG-C-CIP-DXT-GM-NA-NOR-T-TS’ occurred in duplicate. This indicates that all the MDR DEC across the study sites were exposed to unique and diversified pressures selective for the resistance phenotypically observed across the study sites. This is not surprising as surface water bodies are almost always open to sewage and wastewater discharge which may contain partially broken antibiotics, resistant bacteria as well as resistance determinants [49].

**Table 2:** The MARPS patterns of DEC across the selected access points of the Buffalo River.

SN	MAR Phenotypes	No. of Antibiotics	No. of Isolates	MARI
	**Maden dam**			
1	AUG-AP-C-CXM-NI-T	6	1	0.4
2	AUG-AP-GM-T	4	2	0.3
3	DXT-NA-T	3	1	0.2
4	AP-AUG-GM	3	1	0.2
5	AP-AUG-CXM	3	2	0.2
	**Rooikrantz dam**			
1	AP-AUG-C-CTX-CXM- GM-NA-NI-T-TS	10	1	0.6
2	AP-AUG-C-CTX-CXM-DXT-GM-MEM-NA-NI-T-TS	12	1	0.8
3	AP-AUG- CIP-CTX-CXM-DXT-NA-T-TS	9	1	0.6
4	AP-AUG-TS	3	1	0.2
5	AP-AUG-DXT-T-TS	5	1	0.3
6	AP-AUG-C-CTX-CXM-NI-TS	7	1	0.4
7	AUG-DXT-GM-NI	5	1	0.3
	**King William’s Town dam**			
1	AP-AUG-C-CXM-DXT-IMI-NA-NI-T-TS	10	1	0.6
2	AP-AUG-C-CXM-DXT-GM-IMI-NA-NI-T-TS	11	1	0.7
3	AP-AUG-C-DXT-CIP-CXM-IMI-NA-NI-NOR-T-TS	12	1	0.8
4	AP-AUG-C-CXM-IMI-MEM-NA-NI-TS	9	1	0.6
5	AP-AUG-CXM-DXT-GM-IMI-MEM-NA-NI-T-TS	11	1	0.7
6	AP-DXT-CXM-GM-NI-T-TS	7	1	0.4
7	AP-AUG-DXT-CTX-CXM-NI-T-TS	8	1	0.5
8	AP-AUG-DXT-GM-NI-T-TS	7	1	0.4
9	AP-AUG-DXT-NI-T-TS	6	1	0.4
10	AP-T-TS	3	1	0.2
	**Eluxolzweni dam**			
1	AP-AUG-C-DXT-NA-T-TS	7	1	0.4
2	AP-AUG-DXT-C-CIP-GM-NA-NOR-T-TS	10	2	0.6
3	AP-AUG-C-T	4	1	0.3
4	AP-AUG-C-CTX-CXM-GM-NA-NI-TS	9	1	0.6
5	AP-AUG-C-CTX-CXM-GM-MEM-NA-NI-TS	10	1	0.6
6	AP-AUG-CTX-CXM-GM-NA-NI-T-TS	9	1	0.6
7	AP-AUG-CTX-CXM-NI-T	6	1	0.4
8	AP-AUG-CXM-T	4	1	0.3
9	AP-AUG-CXM-NI-T	5	1	0.3
10	AP-AUG-AK-GM-C-CTX-CXM-IMI-MEM-NI-T-TS	12	1	0.8

SN: Serial number, GM-gentamycin, AK-amikacin, AUG- amoxycillin clavulanic acid, AP-ampicillin, IMI-imipenem, MEM-meropenem, CTX-cefotaxime, CXM- cefuroxime, CIP-ciprofloxacin, NOR-norfloxacin, NI-nitrofurantoin, C-chloramphenicol, NA-nalidixic acid, TS-trimethoprim/sulphamethoxazole, T-tetracycline, DXT-doxycycline.

### 3.5 Genotypic AMR characteristics of the DEC in the selected access points of the Buffalo River

#### 3.5.1 Prevalence and co-occurrence patterns of antimicrobial resistance genes in DEC

The ARGs detected in DEC across the selected access points of the Buffalo River include *tet*A and *tet*B tetracycline resistance encoding genes., *sul*I and *sul*II sulphonamide resistance encoding genes., *cat*I and *cat*II phenicol resistance encoding gene., *str*A aminoglycoside resistance encoding genes., *bla*_FOX_ and *bla*_MOX_ plasmid-mediated *Amp*C β-lactam resistance-encoding gene., *bla*_TEM_, *bla*_SHV_, *bla*_VEB_ and *bla*_PER_ ESBLs., and *bla*_IMP_ and *bla*_KPC_ carbapenemases. This shows that ARGs are gradually becoming more diverse and extensive in surface water bodies, and the Buffalo River is representative of the situation.

**Fig 6:**
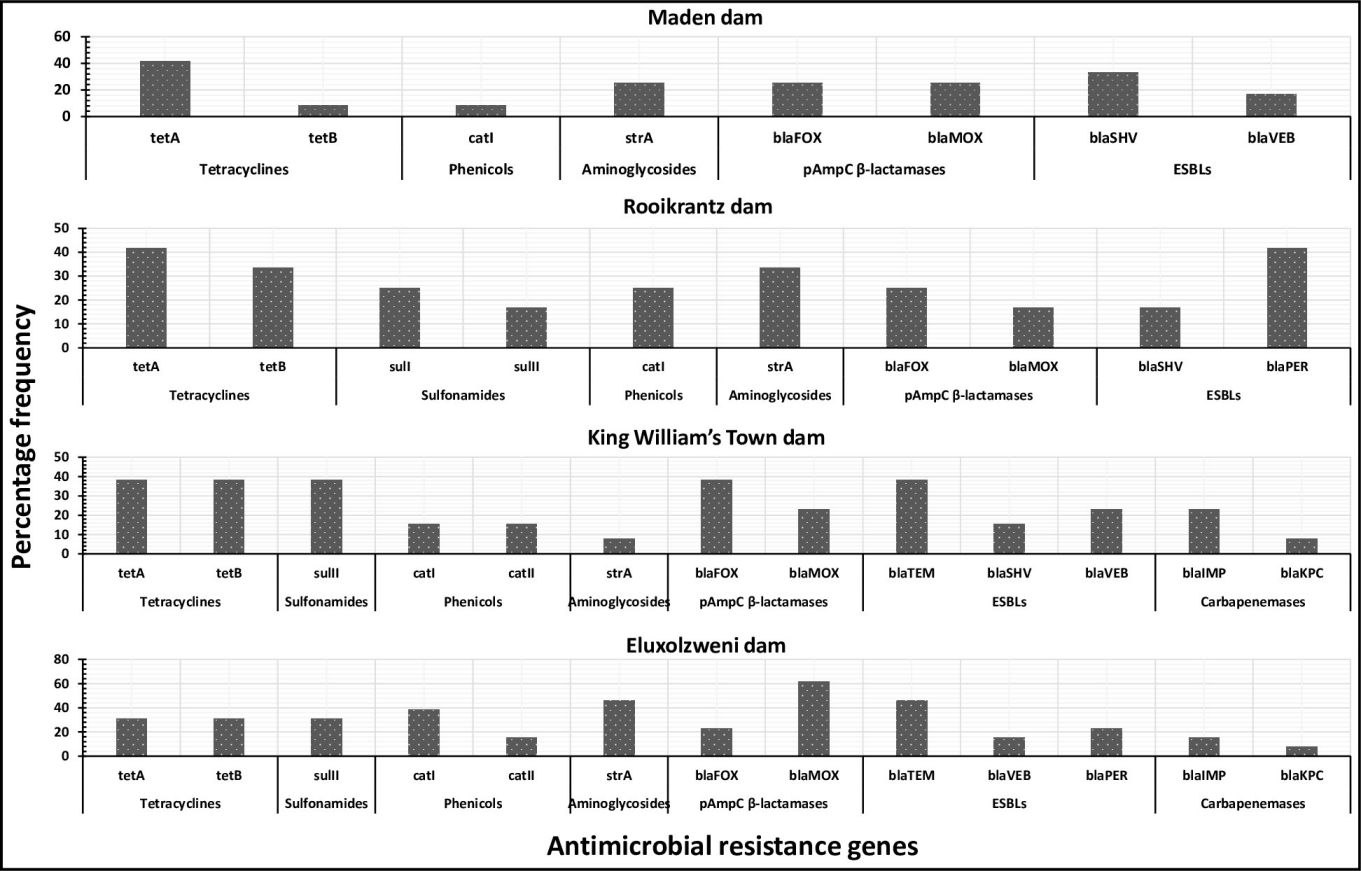
The prevalence of the ARGs in DEC across the selected access points of the Buffalo River.

**Fig 7:**
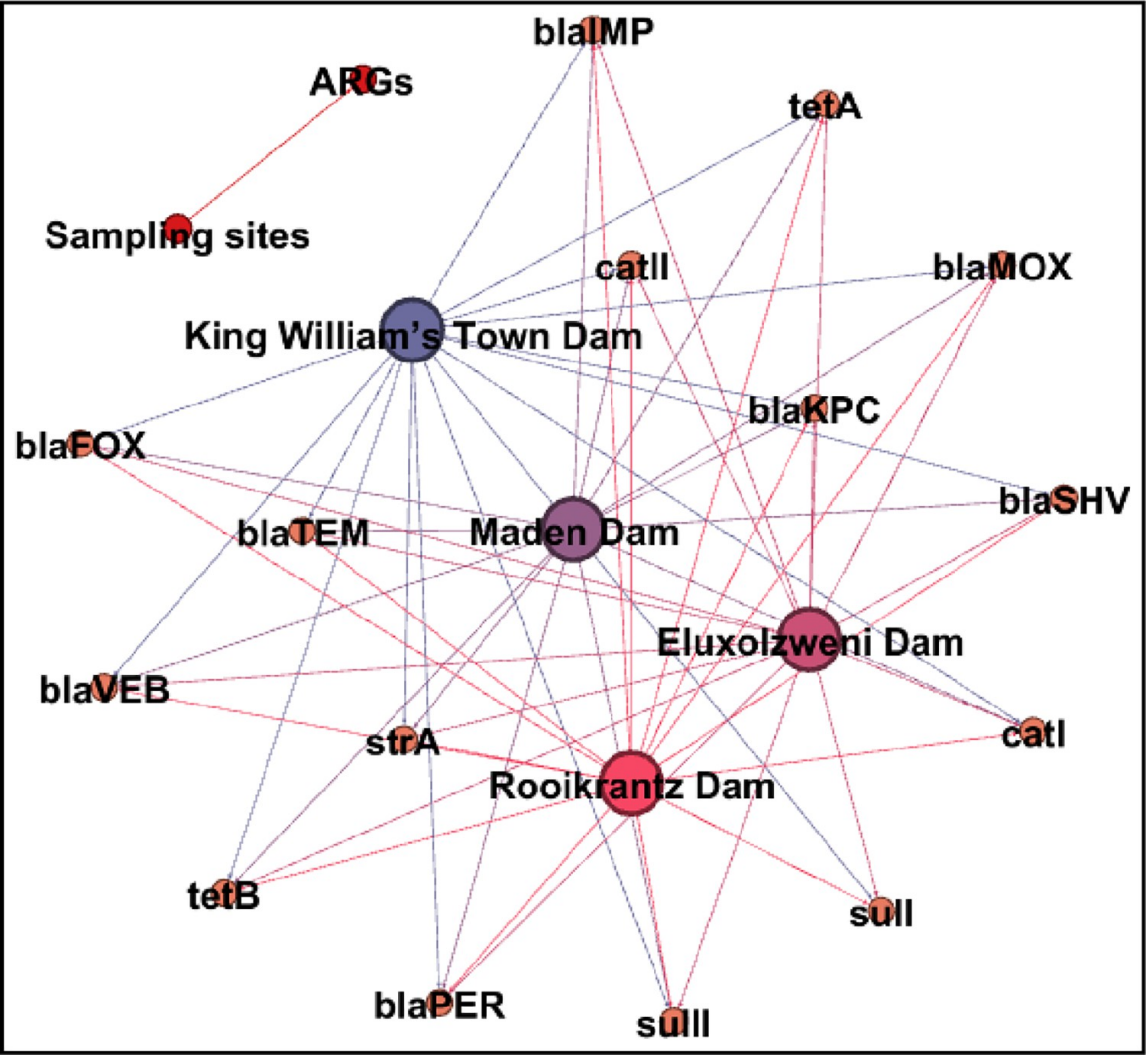
A network analysis plot revealing the co-occurrence patterns of the detected ARGs across the selected access points of the Buffalo River.

The site-specific prevalence of the ARGs in DEC across the selected access points of the Buffalo River is shown in Fig 6. In Maden dam, the prevalence of the ARGs in DEC ranged from 8.3% (*tet*B and *cat*I) to 41.7% (*tet*A), while that in Rooikrantz dam ranged from 16.7% (*sul*II, *bla*_MOX_ and *bla*_SHV_) to 41.7% (*tet*A and *bla*_PER_). In King William’s Town dam, the prevalence of the ARGs in DEC ranged from 7.7% (*str*A and *bla*_KPC_) to 38.5% (*tet*A, *tet*B, *sul*II, *bla*_FOX_, and *bla*_TEM_), while that in Eluxolzweni dam ranged from 7.7% (*bla*_KPC_) to 61.5% (*bla*_MOX_). Of the non β-lactam ARGs/ESBLs, the tetracycline resistance encoding genes particularly the *tet*A were found to be more prevalent across the study sites. The *sul*II sulphonamide resistance encoding gene was also prevalent in King William’s Town dam. This is attributed to the excessive use of tetracyclines and sulphonamides for medical, veterinary and metaphylactic purposes within the catchment of the Buffalo River. The metabolites of these antibiotics eventually find their way to aquatic bodies where they cause selective pressure for the emergence of putative resistance genes [50]. Also, the tetracycline and sulphonamide resistance encoding genes are highly stable and self-amplifying in the aquatic environment [51]. Of the β-lactam ARGs/ESBLs, the *bla*_SHV_ was more prevalent in Maden dam, while the *bla*_TEM_ was more prevalent across the other study sites. This corroborates a similar study whereby *bla*_TEM_ in *E*. *coli* was the dominant ESBL detected in aquatic environments [52]. Also, *bla*_SHV_ and *bla*_TEM_ have previously been found to be dominating in agricultural environments and even clinical settings [53, 54], suggesting that they are widely spread in the ecosystem. There was a significant co-occurrence pattern of the detected ARGs in the selected access points of the Buffalo River as shown in Fig 7. This indicates potential mobility of antimicrobial resistance determinants in different points of the Buffalo River.

#### 3.5.2 Multiple antimicrobial resistance genotypes in DEC

The patterns of the MARGs of the DEC across the selected access points of Buffalo River occurred uniquely and are mapped in Table 3. In the Maden dam, 33% (4/12) of the DEC harboured multiple (≥3) ARGs, all of which were a combination of non β-lactams/ESBLs and β-lactams/ESBLs. In the Rooikrantz dam, 50% (6/12) of the DEC harboured multiple (≥3) ARGs, all of which were a combination of non β-lactams/ESBLs and β-lactams/ESBLs. In King William’s Town dam, 9/13 (69%) of the DEC harboured multiple (≥3) ARGs, all of which were a combination of non β-lactams/ESBLs and β-lactams/ESBLs. In the Eluxolzweni dam, 77% (10/13) of the DEC harboured multiple (≥3) ARGs, the majority of which were a combination of non β-lactams/ESBLs and β-lactams/ESBLs. Only one isolate harboured only β-lactams/ESBLs. This indicates an increase in the dissemination of genetic resistance determinants between antibiotic-resistant bacteria (ARB) and indigenous bacteria of the Buffalo River agreeing to the findings of Araújo et al. (2017) [55]. This may be a result of either horizontal gene transfer or de novo gene mutation consequently leading to altered drug targets, up-regulation of efflux systems (efflux pumps), expression of enzymes capable of inactivating and degrading the antibiotics and loss of uptake mechanisms [56].

**Table 3:** The patterns of MARGs in the DEC recovered across the selected access points of the Buffalo River.

SN	MAR Genotypes	Number of ARGs	Number of non β-lactams and ESBLs	Number of β-lactams and ESBLs	Number of isolates
	**Maden dam**				
1	*bla*_FOX_-*bla*_SHV_-*cat*I-*tet*A	4	2	2	1
2	*bla*_FOX_-*bla*_SHV_-*str*A-*tet*A	4	2	2	1
3	*bla*_MOX_-*bla*_SHV_-*str*A- *tet*A	4	2	2	1
4	*bla*_MOX_-*bla*_VEB_*-str*A	3	1	2	1
	**Rooikrantz dam**				
1	*bla*_FOX_-*bla*_PER_-*sul*I-*str*A*-tet*A	5	3	2	1
2	*bla*_FOX_-*bla*_PER_-*sul*I-*str*A-*tet*A-*tet*B	6	4	2	1
3	*bla*_FOX_-*bla*_PER_-*sul*I-*tet*A-*tet*B	5	3	2	1
4	*bla*_MOX_-*bla*_SHV_-*sul*II-*tet*A-*tet*B	5	3	2	1
5	*bla*_MOX_-*bla*_PER_-*cat*I-*sul*II	4	2	2	1
6	*bla*_PER_-*str*A*-tet*B	3	2	1	1
	**King William’s Town dam**				
1	*bla*_FOX_-*bla*_TEM_-*bla*_IMP_-*cat*II-*sul*II-*tet*A-*tet*B	7	4	3	1
2	*bla*_FOX_-*bla*_SHV_-*bla*_IMP_-*cat*II-*str*A-*sul*II-*tet*A-*tet*B	8	5	3	1
3	*bla*_FOX_-*bla*_SHV_-*bla*_IMP_*-cat*I	4	1	3	1
4	*bla*_FOX_-*bla*_TEM_*-cat*I	3	1	2	1
5	*bla*_MOX_-*bla*_TEM_-*bla*_KPC_*-sul*II	4	1	3	1
6	*bla*_MOX_-*bla*_TEM_*-sul*II	3	1	2	1
7	*bla*_MOX_-*bla*_TEM_*-tet*B	3	1	2	1
8	*bla*_VEB_*-tet*A-*tet*B-*sul*II	4	3	1	1
9	*bla*_VEB_*-tet*A-*tet*B	3	2	1	1
	**Eluxolzweni dam**				
1	*bla*_MOX_-*cat*I-*tet*A-*tet*B-*sul*II	5	4	1	1
2	*bla*_MOX_-*bla*_TEM_-*cat*I*-tet*A-*tet*B -*str*A	6	4	2	1
3	*bla*_MOX_-*bla*_TEM_-*cat*I-*sul*II-*str*A	5	3	2	1
4	*bla*_MOX_-*bla*_TEM_*-cat*I	3	1	2	1
5	*bla*_FOX_-*bla*_VEB_*-cat*II-*str*A	4	2	2	1
6	*bla*_FOX_-*bla*_VEB_-*bla*_IMP_*-cat*II*-sul*II-*str*A	6	3	3	1
7	*bla*_FOX_-*bla*_PER_-*str*A-*tet*B	4	2	2	1
8	*bla*_MOX_-*bla*_TEM_-*bla*_PER_	3	0	3	1
9	*bla*_MOX_-*bla*_TEM_*-tet*A	3	1	2	1
10	*bla*_MOX_-*bla*_TEM_-*bla*_IMP_-*bla*_KPC_*-cat*I-*sul*II-*str*A*-tet*A-*tet*B	9	5	4	1

### 3.6 The association between the phenotypic and genotypic antimicrobial profiles of DEC in each study site

**Fig 8:**
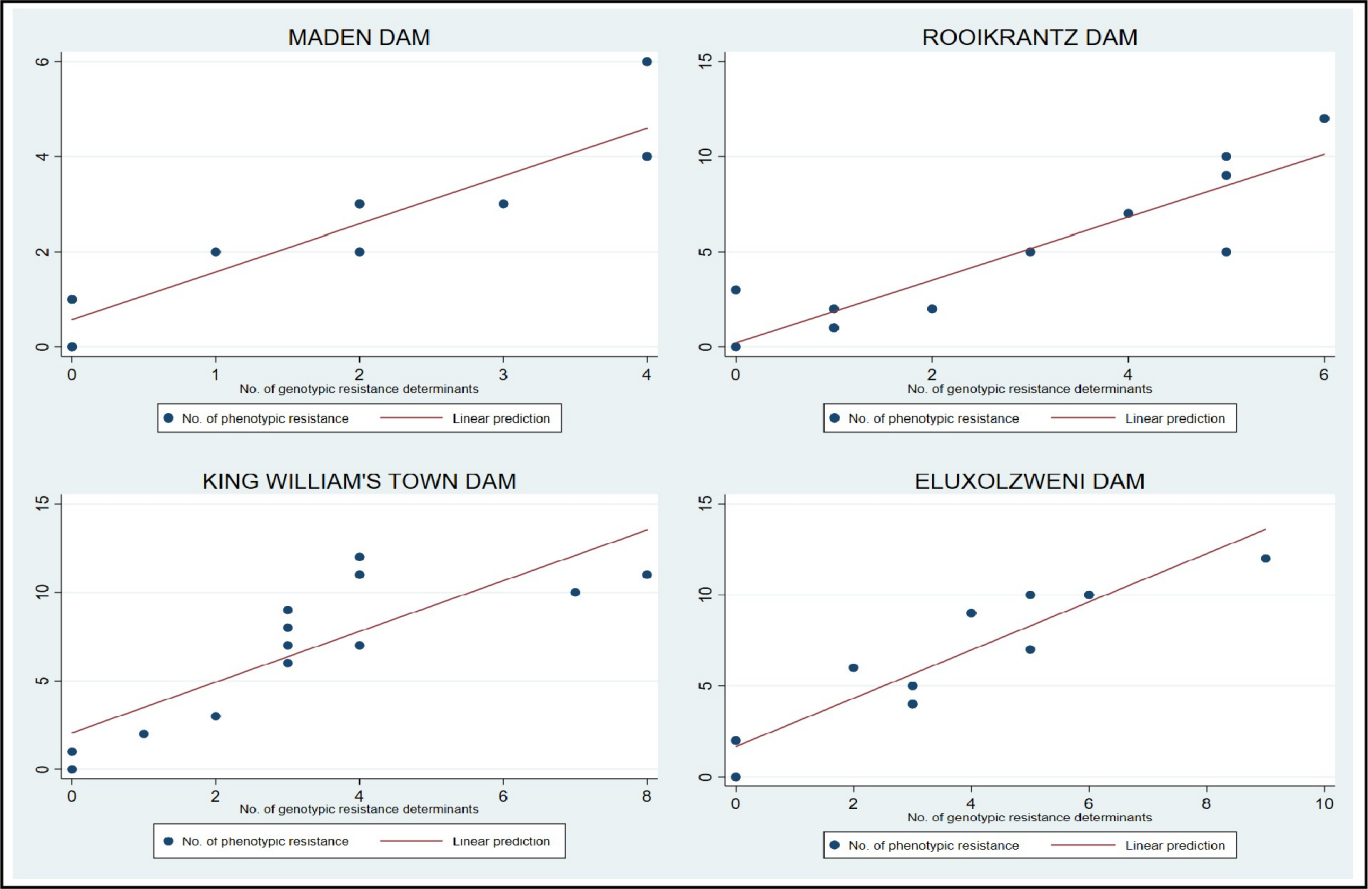
A two-way scatter plot with a linear predictive line showing how correlated the genotypic AMR characteristics is with the phenotypic AMR characteristics of the DEC in each study site.

In this study, a very strong positive correlation existed between the genotypic and phenotypic AMR profiles of the DEC recovered from Maden dam (r = 0.93, *p*<0.00), Rooikrantz dam (r = 0.91, *p*<0.00), King William’s Town dam (r = 0.83, *p =* 0.0), and Eluxolzweni dam (r = 0.91, *p*<0.00). Additionally, there was sufficient evidence that the genotypic AMR characteristics of the DEC significantly predicated the phenotypic AMR characteristics of the DEC in the Maden dam (R^2^ = 0.86, β = 1.01, 95% CI: 0.72 to 1.29, *p*<0.00), Rooikrantz dam (R^2^ = 0.83, β = 1.65, 95% CI: 1.12 to 2.18, *p*<0.00), King William’s Town dam (R^2^ = 0.69, β = 1.43, 95% CI: 0.80 to 2.07, *p*<0.00) and Eluxolzweni dam (R^2^ = 0.83, β = 1.32, 95% CI: 0.92 to 1.73, *p*<0.00) as shown in Fig 8. In Maden dam, Rooikrantz dam, King William’s Town dam and Eluxolzweni dam, it is predicted that every single increase in the number of genotypic resistance determinants in the DEC is associated with a 1.01, 1.65, 1.43 and 1.32 change increase in the number of phenotypic resistances in the DEC within each study site respectively. It, therefore, shows that the observed resistance in this study was acquired. This agrees with a previous study that indicated that an acquired resistance mechanism where a mutation or horizontal gene transfer confers resistance (typically by modifying/degrading the antibiotic or modifying/protecting the drug target) often results in a predictable increase in phenotypic resistance irrespective of the bacterial growth conditions or the genetic context [57].

Based on the R^2^ statistics, 86%, 83%, 69% and 83% of the total variance in the phenotypic AMR characteristics of the DEC in Maden dam, Rooikrantz dam, King William’s Town dam and Eluxozweni dam respectively is explained by the variance of the genotypic AMR characteristics of the isolates in each study site. The R^2^ statistics of the DEC in King William’s Town dam were relatively low compared to that in other study sites. This is because the linear relationship between the phenotypic and genotypic AMR characteristics of the DEC in the study site was not as strong as that in other study sites. This may be attributed to full or partial disconnection of specific resistance phenotypes from the associated genotype. As such, the presence of an ARG/mutation may not necessarily lead to the observed phenotypic resistance. This usually occurs due to (i) environmental factors which alter the phenotypic resistance for a given genotype, (ii) varying genetic context of the resistance determinants which influence the phenotypic resistance of a given ARG or mutation [57].

### 3.7 Antibiogram-based diversities of DEC in the selected access points of the Buffalo River

**Fig 9:**
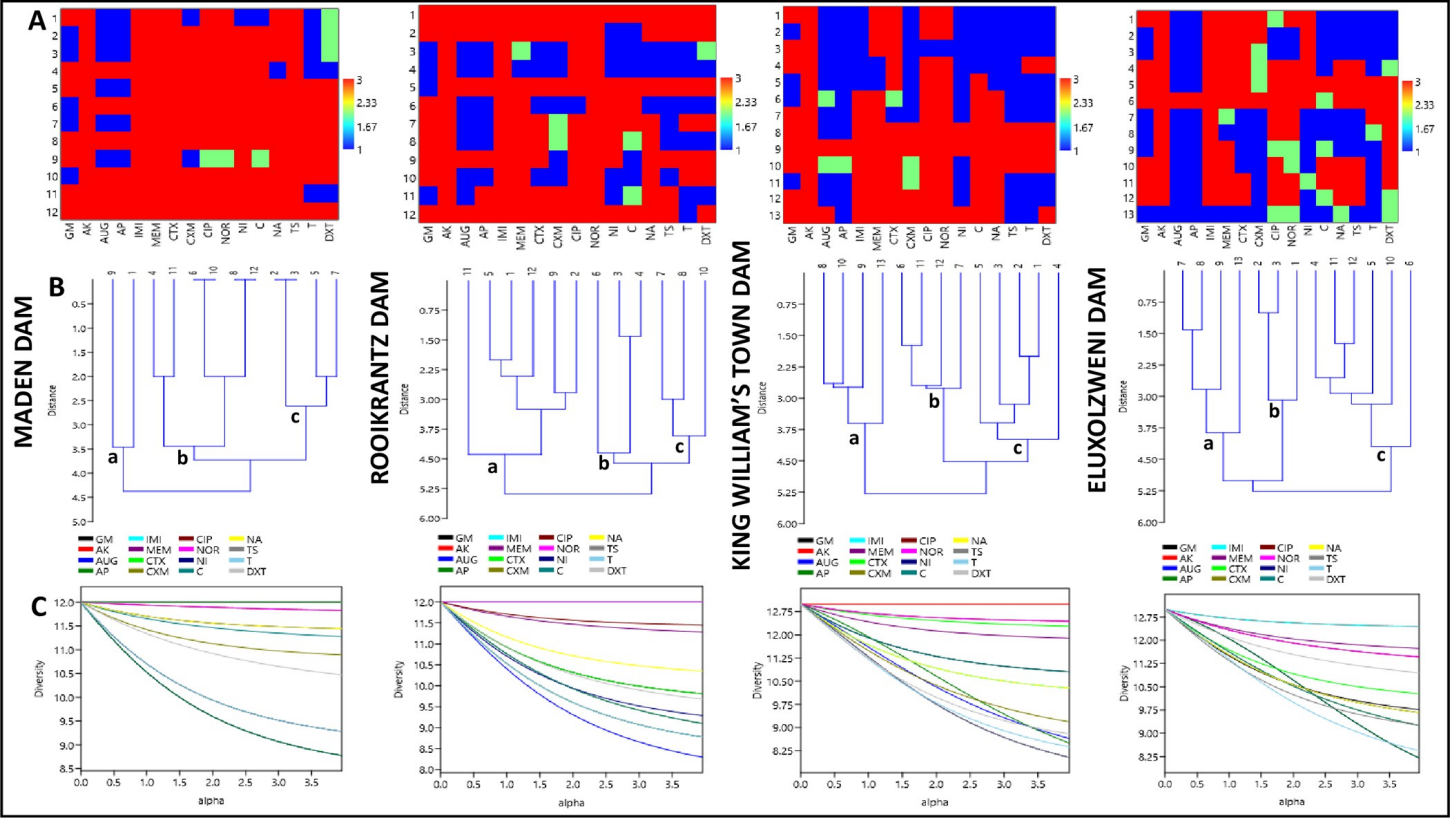
Antibiogram based diversities of DEC in the selected access points of the Buffalo River. (A) Heatmap of the antibiogram fingerprints of the DEC (Red-3: Susceptible, Blue-1: Resistance, Green-2: Intermediate), (B) Neighbor-joining dendrogram clusters of DEC, (C) α-diversity curves of the DEC.

The Antibiogram-based diversities of DEC in the selected access points of the Buffalo River are shown in Fig 9. The heatmaps indicated that the DEC from Eluxolzweni dam were more heterogeneous in their response to the test antimicrobials, followed by that in King William’s Town dam, Rooikrantz dam and Maden dam. This resulted in at least 3 phylogenetic clades (a to c) across the selected access points of the Buffalo River. All the diversity curves across the study sites had a similar shape and portrayed a curvature for most of the test antimicrobials. This indicates that the DEC isolates across the study sites were relatively uneven based on their responses to the test antimicrobials, hence highly diversified [35]. This poses significant environmental and health issues as the DEC with diversified antibiogram characteristics will continue to exchange their genetic materials with related and unrelated bacterial pathogens via horizontal gene transfer.

### 3.8 Study strengths and limitations

To the best of our knowledge, this is the first study that fully characterized the diarrhoeal disease risks and antibiogram diversity of DEC in the Buffalo River. This study employed a combination of standard epidemiological, microbiological, and molecular methods which was robust enough to efficiently answer the research questions. However, the study limitation was ascribed to the grab sampling technique which led to the small sample size, and cross-sectional study design which only provided a snapshot of the diarrhoeal disease risk and antibiogram diversity of DEC across the selected access points of the Buffalo River. Additional studies may be required to account for seasonal variations.

## 4. Conclusion and implications for public health

In conclusion, our study serves as a compelling proof of concept for the application of the stated methods in assessing the risks of diarrheal diseases from waterbodies. The findings highlight the Buffalo River as an emerging reservoir of DEC, which poses diarrhoeal disease risks and exhibits diversified multiple antimicrobial resistance. The isolates haboured clinically significant VGs and ARGs. This constitutes an epidemiological concern, especially to the immunocompromised since the Buffalo River serves as a significant source of water for domestic, agricultural, and recreational purposes in the Eastern Cape Province. Additionally, this poses other health and socio-economic challenges such as increased risks of diarrhoeal disease outbreaks, extra hospitalization stays and treatment costs due to AMR, challenges to researchers and scientists developing new antimicrobials and extra costs to the government trying to address this issue. An urgent intervention involving risk management and measures to control the release of contaminants into aquatic environments is needed.

## Supporting information

S1 File Supplementary tables. (PDF)

S1 Raw images (PDF)
