# Supplementary material for: Spatial diarrheal disease risks and antibiogram diversity of diarrheagenic Escherichia coli in selected access points of the Buffalo River, South Africa
Source: PLoS One. 2023 Aug 24;18(8):e0288809. doi: 10.1371/journal.pone.0288809 (PMC10449160; doi:10.1371/journal.pone.0288809)
Supplement: S1 File — (PDF) [file pone.0288809.s001.pdf]

Supplementary Table 1: List of the four sampling sites, their codes, geographical description and observed anthropogenic activities within the study sites

Site name	Description	Anthropogenic Activities	Coordinates
Maden dam	This dam is about 22km from King William’s Town and is well known for regular fishing activities. The dam is surrounded by weed beds which provide food for the fish.	Fishing, tourism and domestic activities.	32° 44' 00'' S, 27° 16' 00'' E
Rooikrantz dam	It is a dam on the Buffalo River, about 15 km northwest of King William’s Town in the Eastern Cape Province. It lies due east of the larger and newer Sandile Dam.	Fishing, animal rearing, irrigation, tourism and domestic purposes	32° 45' 2'' S, 27° 19' 07'' E
King William's Town	King William's Town is along the banks of the Buffalo River. The area's economy depends on cattle and sheep ranching, and the town itself has a large industrial base producing textiles, soap, candles, sweets, cartons and clothing.	Deposition of dirt and chemical pollutants, dumping site, WWTP effluent discharge	32° 53' S, 27° 24' E
Eluxolzweni	Eluxolzweni is an informal settlement situated next to the N2 freeway just north of Mdantsane. It falls under Buffalo City Metropolitan Municipality in the Eastern Cape Province of South Africa.	Fishing, irrigation, domestic purposes, WWTP effluent discharge	32° 91' 36'' S, 27° 75' 69'' E

WWTP: Wastewater Treatment Plant (Report highlights impacts on river health, 2005).

Supplementary Table 2: Primer sequence, PCR cycling conditions and expected amplicon sizes for the confirmation and delineation of *E. coli* into its pathotypes.

Target strains	Gene	Primer sequence (5′-3′)	PCR cycling conditions	Amplicon size	Reference
E. coli	*uidA* housekeeping gene	F: AAAACGGCAAGAAAAAGCAG<br>R: ACGCGTGGTTAACAGTCTTGCG	94 °C, 5 min; 35[95 °C, 30 sec; 58 °C, 1 min; 72 °C, 1 min] 72 °C, 8 min	147	(Moyo et al., 2007)
EAEC	*eagg* adhesion gene	F: AGACTCTGGCGAAAGACTGTATC<br>R: ATGGCTGTCTGTAATAGATGAGAAC	95 °C, 15 min; 35 cycles [94 °C, 45 s; 55 °C, 45 s; 68 °C, 2 min]; 72 °C, 5 min	194	(Kong et al., 2002)
EIEC	*ipaH* invasion gene	F: CTC GGCACGTTTTAATAGTCTGG<br>R: GTGGAGAGCTGAAGTTTCTCTGC	95 °C, 5 min; 30 cycles [95 °C, 45 sec; 55 °C, 45 sec; 72 °C, 45 sec]; 72 °C, 10 min	320	(Vidal et al., 2005)
DAEC	*daa*E adhesion gene	F: GAACGTTGGTTAATGTGGGGTAA<br>R: TATTCACCGGTCGGTTATCAGT	94 °C, 2 min; 40 cycles [92°C, 30 s; 59 °C, 30 s; 72 °C, 30 s]; 72 °C, 5 min	542	(Vidal et al., 2005)
ETEC	*lt* toxin gene	F: GGCGACAGATTATACCGTGC<br>R: CGGTCTCTATATTCCCTGTT	94 °C, 2 min; 35 cycles [94 °C, 1 min; 55 °C, 1 min, 72 °C, 1 min]; 72 °C, 5 min	450	(López-Saucedo et al., 2003)
EPEC	*eae* adhesion gene	F: TCAATGCAGTTCCGTTATCAGTT<br>R: GTAAAGTCCGTTACCCCAACCTG	95 °C, 15 min; 35 cycles [94 °C, 45 s; 55 °C, 45 s; 68 °C, 2 min]; 72 °C, 5 min	482	(Stacy-Phipps et al., 1995)
	bfp adhesion gene	F: AGACTCTGGCGAAAACTGTATC<br>R: ATGGCTGTCTGTAATAGATGAGAAC	94 °C, 2 min; 40 cycles [94 °C, 1 min; 55 °C, 1 min; 72 °C, 1 min]; 72 °C, 5 min	300	(Stacy-Phipps et al., 1995)
EHEC	*stx1* toxin gene	F: ATAAATCGCCATTCGTTGACTAC<br>R: AGAACGCCCACTGAGATCATC	94 °C, 3 min; 35 cycles [93 °C, 60 s; 55 °C, 60 s; 72 °C, 60 sec]; 72 °C, 7 min	180	(Paton and Paton, 1998)
	stx2 toxin gene	F: GGCACTGTCTGAAACTGCTCC<br>R: TCGCCAGTTATCTGACATTCTG	94 °C, 3 min; 35 cycles [93 °C, 60 s; 55 °C, 60 s; 72 °C, 60 sec]; 72 °C, 7 min	255	(Paton and Paton, 1998)

Supplementary Table 3: Data inputted for exposure and diarrheal disease risk modelling in each study site

	**Maden dam**	**Rooikrantz dam**	**King William's Town dam**	**Eluxolzweni dam**	**Data source**
Concentration (C) of *E. coli* (CFU/100 mls × 10^2)	Min: 9.20<br>Mean: 9.53<br>Max: 9.80	Min: 10.80<br>Mean: 11.00<br>Max: 11.20	Min: 15.00<br>Mean: 16.00<br>Max: 17.00	Min: 7.00<br>Mean: 8.17<br>Max: 9.50	This study
Recovery efficiency (R) (%)	16.00	12.00	4.00	8.00	This study
Proportion (I) of *E. coli* capable of causing diarrheal disease (%)	29.00	27.00	27.00	28.00	This study
Daily amount (M) of water directly or indirectly ingested (ml/day)	10.00	10.00	10.00	10.00	(Shuval et al., 1997)

Min: Minimum, Max: Maximum

Supplementary Table 4: list of antimicrobials used to carry out antimicrobial susceptibility testing of the DEC

SN	Antimicrobial	Code/concentration	Class
1	Doxycycline	DXT/30 µg	Tetracyclines
2	Tetracycline	T/30 µg	
3	Norfloxacin	NOR/30 µg	Fluoroquinolones
4	Ciprofloxacin	CIP/5 µg	
5	Chloramphenicol	C/30 µg	Phenicols
6	Nalidixic acid	NA/30 µg	Quinolones
7	Cefuroxime	CXM/30 µg	Cephems
8	Cefotaxime	CTX/30 µg	
9	Trimethoprim/sulphamethoxazole	TS/25 µg:25 µg	Sulfonamides
10	Amikacin	AK/30 µg,	Aminoglycosides
11	Gentamycin	GM/10 µg	
12	Nitrofurantoin	NI/300 µg	Nitrofurans
13	Ampicillin	AP/10 µg	β-lactams
14	Amoxicillin-clavulanic acid	AUG/30 µg	
15	Meropenem	MEM/10 µg	Carbapenems
16	Imipenem	IMI/10 µg	

Supplementary Table 5: The primer sequence and expected amplicon size used for the screening of resistance genes in the pathogenic strains of *E. coli*

**Antibiotic class**	**Genes**	**PCR primer sequence (5′–3′)**	**PCR cycling condition**	**Amplicon size (bp)**	**Reference**
Tetracyclines	*tet*A	F: GCTACATCCTGCTTGCCTTC<br>R: CATAGATCGCCGTGAAGAGG	94 °C,5m; 35[94 °C,1m; 55 °C,1m; 72 °C,1.5m]; 72 °C,5m	210	(Mendez et al., 1980)
	*tet*B	F: TTGGTTAGGGGCAAGTTTTG<br>R: GTAATGGGCCAATAACACCG	94 °C,5m; 35[94 °C,1m; 55 °C,1m; 72 °C,1.5m]; 72 °C,5m	659	(Marshall et al., 1983)
	*tet*C	F: CTTGAGAGCCTTCAACCCAG<br>R: ATGGTCGTCATCTACCTGCC	94 °C,5m; 35[94 °C,1m; 55 °C,1m; 72 °C,1.5m]; 72 °C,5m	418	(Marshall et al., 1983)
	*tet*D	F: AAACCATTACGGCATTCTGC<br>R: GACCGGATACACCATCCATC	94 °C,5m; 35[94 °C,1m; 55 °C,1m; 72 °C,1.5m]; 72 °C,5m	787	(Marshall et al., 1983)
	*tet*E	F: AAACCACATCCTCCATACGC<br>R: AAATAGGCCACAACCGTCAG	94 °C,5m; 35[94 °C,1m; 55 °C,1m; 72 °C,1.5m]; 72 °C,5m	278	(Marshall et al., 1986)
	*tet*G	F: GCTCGGTGGTATCTCTGCTC<br>R: AGCAACAGAATCGGGAACAC	94 °C,5m; 35[94 °C,1m; 55 °C,1m; 72 °C,1.5m]; 72 °C,5m	468	(Zhao and Aoki, 1992)
	*tet*K	F: TCGATAGGAACAGCAGTA<br>R: CAGCAGATCCTACTCCTT	94 °C,5m; 35[94 °C,1m; 55 °C,1m; 72 °C,1.5m]; 72 °C,5m	169	(Warsa et al., 1996)
	*tet*L	F: TCGTTAGCGTGCTGTCATTC<br>R: GTATCCCACCAATGTAGCCG	94 °C,5m; 35[94 °C,1m; 55 °C,1m; 72 °C,1.5m]; 72 °C,5m	267	(Burdett et al., 1982)
	*tet*M	F: GTGGACAAAGGTACAACGAG<br>R: CGGTAAAGTTCGTCACACAC	94 °C,5m; 35[94 °C,1m; 55 °C,1m; 72 °C,1.5m]; 72 °C,5m	406	(Warsa et al., 1996)

Sulfonamides	*sulI*	F: CGGCGTGGGCTACCTGAACG<br>R: GCCGATCGCGTGAAGTTCCG	94 °C,5m; 30[94 °C,30s; 65 °C,30s; 72 °C,2m]; 72 °C,10m	433	(Kerrn et al., 2002)
	sulII	F: GCGCTCAAGGCAGATGGCATT<br>R: GCGTTTGATACCGGCACCCGT	94 °C,5m; 30[94 °C,30s; 65 °C,30s; 72 °C,2m]; 72 °C,10m	293	(Kerrn et al., 2002)
Phenicols	*cmlA1*	F: CACCAATCATGACCAAG<br>R: GGCATCACTCGGCATGGACATG	94 °C,5m; 30[94 °C,30s; 50 °C,30s; 72 °C,1.5m]; 72 °C,5m	115	(Post and Hall, 2009)
	catI	F: AGTTGCTCAATGTACCTATAACC<br>R: TTGTAATTCATTAAGCATTCTGCC	94 °C,5m; 30[94 °C,30s; 50 °C,30s; 72 °C,1.5m]; 72 °C,5m	320	(Maynard et al., 2004)
	catII	F: ACACTTTGCCCTTTATCGTC<br>R: TGAAAGCCATCACATACTGC	94 °C,5m; 30[94 °C,30s; 50 °C,30s; 72 °C,1.5m]; 72 °C,5m	543	(Maynard et al., 2004)
Aminoglycosides	*strA*	F CTTGGTGATAACGGCAATTC<br>R: CCAATCGCAGATAGAAGGC	94 °C,4m; 30[94 °C,45s; 50 °C,45s; 72 °C,45s]; 72 °C,5m	348	(Velusamy et al., 2007)
	aadA	F: GTGGATGGCGGCCTGAAGCC<br>R: AATGCCCAGTCGGCAGCG	94 °C,4m; 30[94 °C,45s; 50 °C,45s; 72 °C,45s]; 72 °C,5m	525	(Velusamy et al., 2007)
	aac(3)-IIa (aacC2)[a]	F: CGGAAGGCAATAACGGAG<br>R: TCGAACAGGTAGCACTGAG	94 °C,5m; 30[94 °C,30s; 50 °C,30s; 72 °C,1.5m]; 72 °C,5m	428	(Maynard et al., 2004)
	aph(3)-Ia (aphA1)[a]	F: ATGGGCTCGCGATAATGTC<br>R: CTCACCGAGGCAGTTCCAT	94 °C,5m; 30[94 °C,30s; 50 °C,30s; 72 °C,1.5m]; 72 °C,5m	600	(Maynard et al., 2004)

	aph(3)-IIa (aphA2)[a]	F: GAACAAGATGGATTGCACGC<br>R: GCTCTTCAGCAATATCACGG	94 °C,5m; 30[94 °C,30s; 50 °C,30s; 72 °C,1.5m]; 72 °C,5m	510	(Maynard et al., 2004)

Supplementary Table 6: The primer sequence and expected amplicon size used for the screening of *AmpC* β-lactamase (Velusamy et al., 2007) and ESBLs in pathogenic strains of *E. coli* (Dallenne et al., 2010).

**PCR name**	**Targeted β-Lactamase(s)**	**Primer name**	**Primer sequence (5' –3')**	**PCR cycling condition**	**Amplicon size (bp)**
Simplex AmpC[a]	*AmpC*	AmpC_for<br>AmpC_rev	TTCTATCAAMACTGGCARCC<br>CCYTTTTATGTACCCAYGA	94 °C, 4m; 30[94 °C, 45 s; 60 °C, 45s; 72 °C, 45s]; 72 °C, 7m	550
Multiplex I TEM, SHV and OXA-1-like	TEM variants including TEM-1 and TEM-2	MultiTSO-T_for<br>MultiTSO-T_rev	CATTTCCGTGTCGCCCTTATTC<br>CGTTCATCCATAGTTGCCTGAC	94 °C, 10m; 30[94 °C, 40 s; 60 °C, 40s; 72 °C, 1m]; 72 °C, 7m	800
	SHV variants including SHV-1	MultiTSO-S_for<br>MultiTSO-S_rev	AGCCGCTTGAGCAAATTAAAC<br>ATCCCGCAGATAAATCACCAC		713
	OXA-1, OXA-4 and OXA-30	MultiTSO-O_for<br>MultiTSO-O_rev	GGCACCAGATTCAACTTTCAAG<br>GACCCCAAGTTTCCTGTAAGTG		564
Multiplex II CTX-M group 1, group 2 and group 9	Variants of CTX-M group 1 including CTX-M-1, CTX-M-3 and CTX-M-15	MultiCTXMGp1_for<br>MultiCTXMGp1-2_rev	TTAGGAARTGTGCCGCTGYA[b]<br>CGATATCGTTGGTGGTRCCAT[b]	94 °C, 10m; 30[94 °C, 40 s; 60 °C, 40s; 72 °C, 1m]; 72 °C, 7m	688
	variants of CTX-M group 2 including CTXM-2	MultiCTXMGp2_for<br>MultiCTXMGp1-2_rev	CGTTAACGGCACGATGAC<br>CGATATCGTTGGTGGTRCCAT[b]		404
	Variants of CTX-M group 9 including CTX-M-9 and CTX-M-14	MultiCTXMGp9_for<br>MultiCTXMGp9_rev	TCAAGCCTGCCGATCTGGT<br>TGATTCTCGCCGCTGAAG		561
CTX-M group 8/25	CTX-M-8, CTX-M-25, CTX-M-26 and CTX-M-39 to CTX-M-41	CTX-Mg8/25_for<br>CTX-Mg8/25_rev	AACRCRCAGACGCTCTAC[b]<br>TCGAGCCGGAASGTGTYAT[b]		326

Multiplex III ACC, FOX, MOX, DHA, CIT and EBC (plasmid-mediated AmpC)	ACC-1 and ACC-2	MultiCaseACC_for<br>MultiCaseACC_rev	CACCTCCAGCGACTTGTTAC<br>GTTAGCCAGCATCACGATCC	94 °C, 10m; 30[94 °C, 40 s; 60 °C, 40s; 72 °C, 1m]; 72 °C, 7m	346
	FOX-1 to FOX-5	MultiCaseFOX_for<br>MultiCaseFOX_rev	CTACAGTGCGGGTGGTTT<br>CTATTTGCGGCCAGGTGA		162
	MOX-1, MOX-2, CMY-1, CMY-8 to CMY-11 and CMY-19	MultiCaseMOX_for<br>MultiCaseMOX_rev	GCAACAACGACAATCCATCCT<br>GGGATAGGCGTAACTCTCCCAA		895
	DHA-1 and DHA-2	MultiCaseDHA_for<br>MultiCaseDHA_rev	TGATGGCACAGCAGGATATTC<br>GCTTTGACTCTTTCGGTATTCG		997
	LAT-1 to LAT-3, BIL-1, CMY-2 to MY-7, CMY-12 to CMY-18 and CMY-21 to CMY-23	MultiCaseCIT_for<br>MultiCaseCIT_rev	CGAAGAGGCAATGACCAGAC<br>ACGGACAGGGTTAGGATAGY[b]		538
	ACT-1 and MIR-1	MultiCaseEBC_for<br>MultiCaseEBC_rev	CGGTAAAGCCGATGTTGCG<br>AGCCTAACCCCTGATACA		683
Multiplex IV VEB, PER and GES	GES-1 to GES-9 and GES-11	MultiGES_for<br>MultiGES_rev	AGTCGGCTAGACCGGAAAG<br>TTTGTCCGTGCTCAGGAT	94 °C, 10m; 30[94 °C, 40 s; 57 °C, 40s; 72 °C, 1m]; 72 °C, 7m	399
	PER-1 and PER-3	MultiPER_for<br>MultiPER_rev	GCTCCGATAATGAAAGCGT<br>TTCGGCTTGACTCGGCTGA		520
	VEB-1 to VEB-6	MultiVEB_for<br>MultiVEB_rev	CATTTCCCGATGCAAAGCGT<br>CGAAGTTTCTTTGGACTCTG		648

Multiplex V GES and OXA-48-like	GES-1 to GES-9 and GES-11	MultiGES_for<br>MultiGES_rev	AGTCGGCTAGACCGGAAAG<br>TTTGTCCGTGCTCAGGAT	94 °C, 10m; 30[94 °C, 40 s; 60 °C, 40s; 72 °C, 1m]; 72 °C, 7m	399
	OXA-48-like	MultiOXA-48_for<br>MultiOXA-48_rev	GCTTGATCGCCCTCGATT<br>GATTTGCTCCGTGGCCGAAA		281
Multiplex VI IMP, VIM and KPC	IMP variants except IMP-9, IMP-16, IMP-18, IMP-22 and IMP-25	MultiIMP_for<br>MultiIMP_rev	TTGACACTCCATTTACDG[b]<br>GATYGAGAATTAAGCCACYCT[b]	94 °C, 10m; 30[94 °C, 40 s; 55 °C, 40s; 72 °C, 1m]; 72 °C, 7m	139
	VIM variants including VIM-1 and VIM-2	MultiVIM_for[c]<br>MultiVIM_rev[c]	GATGGTGTTTGGTCGCATA<br>CGAATGCGCAGCACCAG		390
	KPC-1 to KPC-5	MultiKPC_for<br>MultiKPC_rev	CATTCAAGGGCTTTCTTGCTGC<br>ACGACGGCATAGTCATTTGC		538

[a] This primer pair was previously described by (Velusamy et al., 2007).

[b]Y=T or C; R=A or G; S=G or C; D=A or G or T.

[c]This primer pair was previously described by (Ellington et al., 2007).

References

Burdett, V., Inamine, J., and Rajagopalan, S. (1982). Heterogeneity of tetracycline resistance determinants in Streptococcus. *J. Bacteriol.* 149, 995–1004. Available at: http://www.ncbi.nlm.nih.gov/pubmed/6801018 [Accessed April 10, 2019].

Dallenne, C., da Costa, A., Decré, D., Favier, C., and Arlet, G. (2010). Development of a set of multiplex PCR assays for the detection of genes encoding important β-lactamases in Enterobacteriaceae. *J. Antimicrob. Chemother.* 65, 490–495. doi:10.1093/jac/dkp498.

Ellington, M. J., Kistler, J., Livermore, D. M., and Woodford, N. (2007). Multiplex PCR for rapid detection of genes encoding acquired metallo-beta-lactamases. *J. Antimicrob. Chemother.* 59, 321–322. doi:10.1093/jac/dkl481.

Kerrn, M. B., Klemmensen, T., Frimodt-Møller, N., and Espersen, F. (2002). Susceptibility of Danish Escherichia coli strains isolated from urinary tract infections and bacteraemia, and distribution of sul genes conferring sulphonamide resistance. *J. Antimicrob. Chemother.* 50, 513–516. doi:10.1093/jac/dkf164.

Kong, R., Lee, S., Law, T., Law, S., and Wu, R. (2002). Rapid detection of six types of bacterial pathogens in marine waters by multiplex PCR. *Water Res.* 36, 2802–2812. doi:10.1016/S0043-1354(01)00503-6.

López-Saucedo, C., Cerna, J. F., Villegas-Sepulveda, N., Thompson, R., Velazquez, F. R., Torres, J., et al. (2003). Single Multiplex Polymerase Chain Reaction To Detect Diverse Loci Associated with Diarrheagenic Escherichia coli. *Emerg. Infect. Dis.* 9, 127–131. doi:10.3201/eid0901.010507.

Marshall, B., Morrissey, S., Flynn, P., and Levy, S. B. (1986). A new tetracycline-resistance determinant, class E, isolated from Enterobacteriaceae. *Gene* 50, 111–117. doi:10.1016/0378-1119(86)90315-X.

Marshall, B., Tachibana, C., and Levy, S. B. (1983). Frequency of tetracycline resistance determinant classes among lactose-fermenting coliforms. *Antimicrob. Agents Chemother.* 24, 835–40. doi:10.1128/AAC.24.6.835.

Maynard, C., Bekal, S., Sanschagrin, F., Levesque, R. C., Brousseau, R., Masson, L., et al. (2004). Heterogeneity among Virulence and Antimicrobial Resistance Gene Profiles of Extraintestinal Escherichia coli Isolates of Animal and Human Origin. *J. Clin. Microbiol.* 42, 5444–5452. Available at: http://www.ncbi.nlm.nih.gov/pubmed/15583263 [Accessed January 21, 2018].

Mendez, B., Tachibana, C., and Levy, S. B. (1980). Heterogeneity of tetracycline resistance determinants. *Plasmid* 3, 99–108. doi:10.1016/0147-619X(80)90101-8.

Moyo, S. J., Maselle, S. Y., Matee, M. I., Langeland, N., and Mylvaganam, H. (2007). Identification of diarrheagenic Escherichia coli isolated from infants and children in Dares Salaam, Tanzania. *BMC Infect. Dis.* 7, 92. doi:10.1186/1471-2334-7-92.

Paton, A. W., and Paton, J. C. (1998). Detection and Characterization of Shiga Toxigenic Escherichia coli by Using Multiplex PCR Assays for stx 1, stx 2, eaeA, Enterohemorrhagic E. coli hlyA, rfb O111, and rfb O157. *J. Clin. Microbiol.* 36, 598–602.

Post, V., and Hall, R. M. (2009). AbaR5, a large multiple-antibiotic resistance region found

in Acinetobacter baumannii. *Antimicrob. Agents Chemother.* 53, 2667–71. doi:10.1128/AAC.01407-08.

Report highlights impacts on river health (2005). Urban development and dense human settlements are impacting, in some cases severely, on the main river system of the Eastern Cape. This is the main finding of the latest State-of-Rivers (SoR) report, which studied the Buffalo River.

Shuval, H., Lampert, Y., and Fattal, B. (1997). Development of a risk assessment approach for evaluating wastewater reuse standards for agriculture. *Water Sci. Technol.* 35, 15–20. doi:10.1016/S0273-1223(97)00228-X.

Stacy-Phipps, S., Mecca, J. J., and Weiss, J. B. (1995). Multiplex PCR assay and simple preparation method for stool specimens detect enterotoxigenic Escherichia coli DNA during course of infection. *J. Clin. Microbiol.* 33, 1054–9. Available at: http://www.ncbi.nlm.nih.gov/pubmed/7615704 [Accessed January 21, 2018].

Velusamy, S., Gillespie, B. E., Lewis, M. J., Nguyen, L. T., Headrick, S. I., Schukken, Y. H., et al. (2007). Phenotypic and genotypic antimicrobial resistance patterns of Escherichia coli isolated from dairy cows with mastitis. *Vet. Microbiol.* 124, 319–328. doi:10.1016/j.vetmic.2007.04.040.

Vidal, M., Kruger, E., Durán, C., Lagos, R., Levine, M., Prado, V., et al. (2005). Single multiplex PCR assay to identify simultaneously the six categories of diarrheagenic Escherichia coli associated with enteric infections. *J. Clin. Microbiol.* 43, 5362–5. doi:10.1128/JCM.43.10.5362–5365.2005.

Warsa, U. C., Nonoyama, M., Ida, T., Okamoto, R., Okubo, T., Shimauchi, C., et al. (1996). Detection of tet(K) and tet(M) in Staphylococcus aureus of Asian countries by the polymerase chain reaction. *J. Antibiot. (Tokyo).* 49, 1127–32. doi:10.7164/antibiotics.49.1127.

Zhao, J., and Aoki, T. (1992). Nucleotide Sequence Analysis of the Class G Tetracycline Resistance Determinant from Vibrio anguillarum. *Microbiol. Immunol.* 36, 1051–1060. doi:10.1111/j.1348-0421.1992.tb02109.x.
