# Supplementary material for: Spatial diarrheal disease risks and antibiogram diversity of diarrheagenic Escherichia coli in selected access points of the Buffalo River, South Africa
Source: PLoS One. 2023 Aug 24;18(8):e0288809. doi: 10.1371/journal.pone.0288809 (PMC10449160; doi:10.1371/journal.pone.0288809)
Supplement: S1 Raw images — (PDF) [file pone.0288809.s002.pdf]

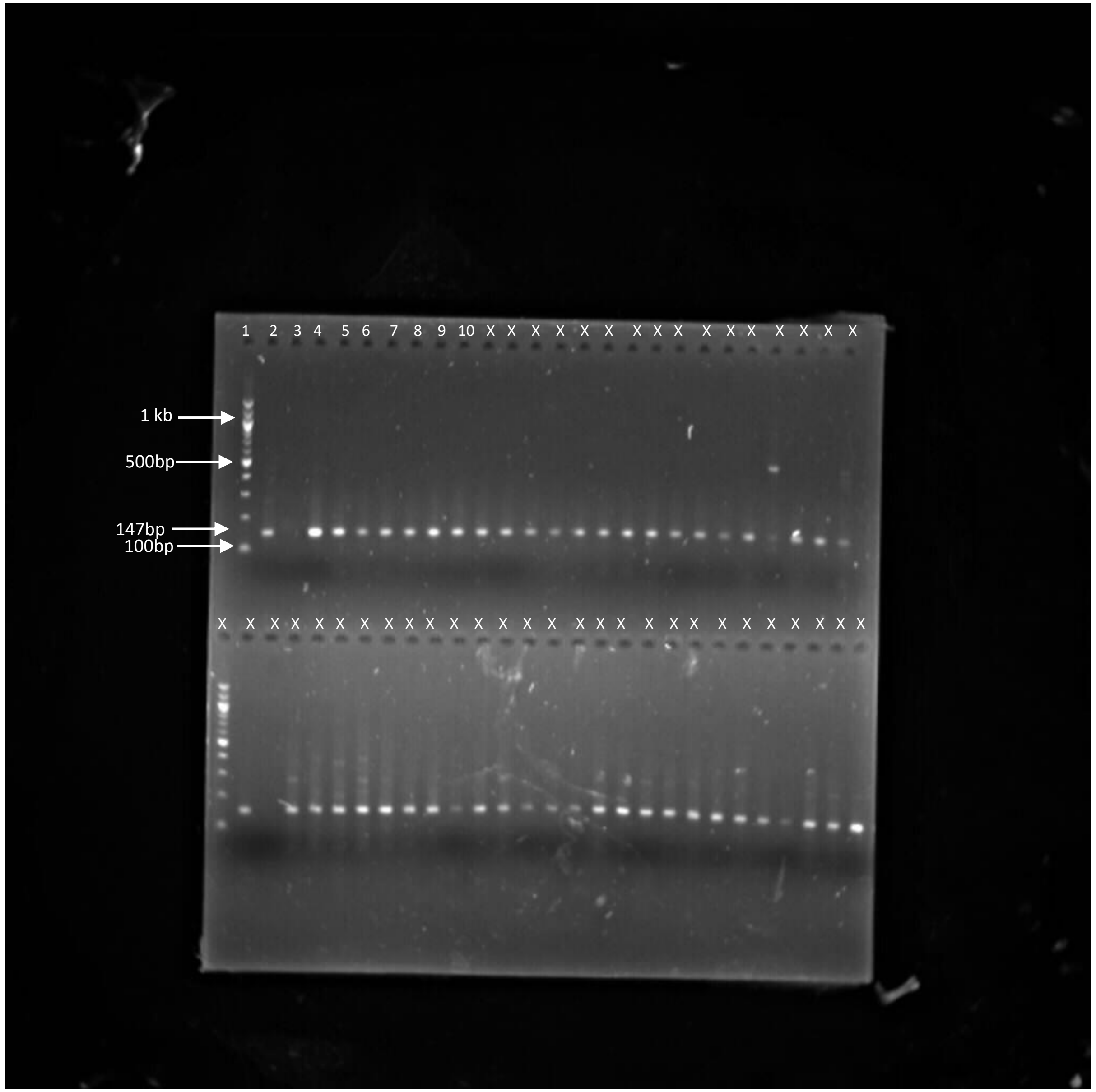

Loading order and experimental samples: Lane 1 on the gel image represents a 100 bp DNA ladder/molecular weight marker, lane 2 represents a positive control (*E. coli* ATCC 25922), lane 3 represent a negative control, lanes 4 to 10 represent positive isolates, and lanes X are lanes not included in the Final Figure

Method used to capture the image: All the PCR products were electrophoresed and captured using the ultraviolet trans-illuminator (Alliance 4.7, United States).

Figure panel that was generated from the original image: Figure 2A.
